# Synthetic study toward tridachiapyrone B

**DOI:** 10.3762/bjoc.18.183

**Published:** 2022-12-19

**Authors:** Morgan Cormier, Florian Hernvann, Michaël De Paolis

**Affiliations:** 1 COBRA, Normandie University, 76000 Rouen, France

**Keywords:** α’-methoxy-γ-pyrone, 2,5-cyclohexadienone, oxy-Cope, quaternary carbon, Robinson-type annulation

## Abstract

A convergent approach to the skeleton of tridachiapyrone B is described taking advantage of the desymmetrization of α,α’-dimethoxy-γ-pyrone leading to α-crotyl-α’-methoxy-γ-pyrone in one step. To construct the quaternary carbon of the 2,5-cyclohexadienone of the target, a strategy based on the Robinson-type annulation of an aldehyde derived from α-crotyl-α’-methoxy-γ-pyrone was applied. The grafting of the simplified target’s side chain was demonstrated through an oxidative anionic oxy-Cope rearrangement of the tertiary alcohol arising from the 1,2-addition of a 1,3-dimethylallyl reagent to 2,5-cyclohexadienone connected to the α’-methoxy-γ-pyrone motif.

## Introduction

**Scheme 1:**
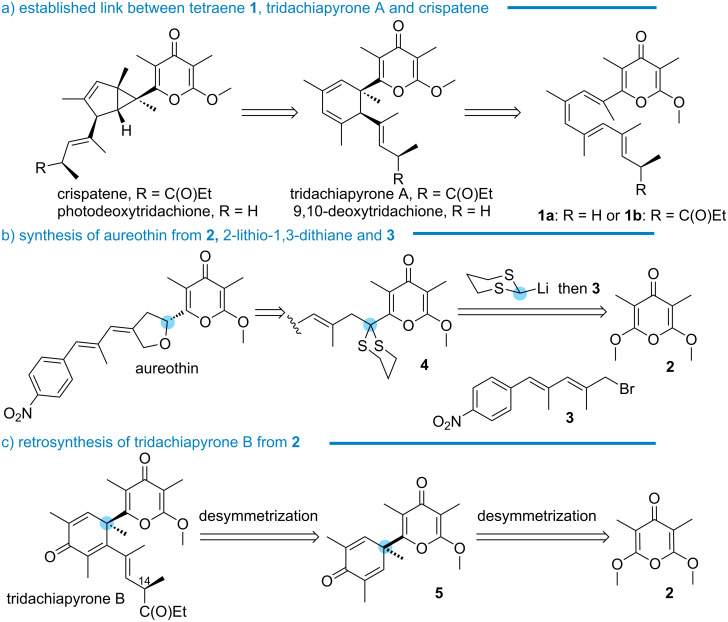
Routes to crispatene, photodeoxytridachione, aureothin, and tridachiapyrone B.

The α’-methoxy-γ-pyrone motif is present in natural products and bioactive molecules [1–14]. Amidst these targets, a number contains a quaternary carbon vicinal to the scaffold, such as crispatene and photodeoxytridachione (Scheme 1a) [15]. These molecules feature a bicyclo[3.1.0]hexene core which photochemically arises from 1,3-cyclohexadiene precursors, tridachiapyrone A or 9,10-deoxytridachione, as demonstrated by Ireland [16–17]. In turn, the ring system arises from α-tetraenyl-α’-methoxy-γ-pyrone precursor **1a** upon heating through 6π-electrocyclization, as illustrated by Baldwin in the synthesis of 9,10-deoxytridachione [18]. In a further demonstration of the versatility of tetraenes connected to α’-methoxy-γ-pyrone, the synthesis of both crispatene and photodeoxytridachione was accomplished by Trauner through the Lewis acid-catalyzed 6π-disrotatory electrocyclization of compounds **1a** and **b** [19–22]. Interestingly, Baldwin and Moses demonstrated the irradiation or sunlight-promoted cycloisomerization of a similar tetraenyl framework into the bicyclo[3.1.0]hexane core through a 6π-conrotatory stereocontrol [23–24].

To date, the known strategies to install a quaternary carbon center connected to α’-methoxy-γ-pyrone therefore rely exclusively on the electrocyclization of tetraenes. With recently demonstrated potent antitumoral [25] and anti-HIV properties [26], aureothin is a natural product featuring the α’-methoxy-γ-pyrone motif connected to a chiral tetrahydrofuran (Scheme 1b). To assemble the skeleton of the natural product, we developed a new strategy in which the α,α’-dimethoxy-γ-pyrone motif **2** was first desymmetrized by a sequence encompassing the conjugate addition of 2-lithio-1,3-dithiane, elimination of methoxide lithium, and deprotonation of 2-(α’-methoxy-γ-pyrone)-1,3-dithiane. The resulting vinylogous enolate intermediate was trapped with the electrophile **3**, amounting to the one-pot preparation of compound **4**, having a masked carbonyl function connecting both key fragments [27–28].

Isolated and characterized by Schmitz [17], the metabolite tridachiapyrone B is related to tridachiapyrone A (Scheme 1c). As the 1,3-cyclohexadiene motif of the latest is oxidized into 2,5-cyclohexadienone, it is assumed that tridachiapyrone B arises from the ring opening of the epoxide (tridachiapyrone C) of tridachiapyrone A. To our knowledge, the synthesis of tridachiapyrone B was not investigated, even though it seems conceivable that the compound’s stability to light, air or cytochrome would be higher than its 1,3-cyclohexadiene counterpart. As the molecule combines electron acceptor functions such as α’-methoxy-γ-pyrone and 2,5-cyclohexadienone, these considerations are advantageous with a view to evaluate the potential activity of tridachiapyrone B and analogues in biological electron transport processes.

With this context in mind, we sought to establish a straightforward access to the key 2,5-cyclohexadienone core connected to α’-methoxy-γ-pyrone by desymmetrization of α,α’-dimethoxy-γ-pyrone **2** through the addition of hindered nucleophiles to construct the vicinal quaternary carbon. In a subsequent and potentially enantioselective desymmetrization step, compound **5** would be converted into trichiachiapyrone B by 1,4-addition of the side chain to the 2,5-cyclohexadienone scaffold. Avoiding heat and light sensitive tetraenes, the convergent plan would also give the opportunity to assess an enantioselective synthesis of the targets, noting that the C^14^ epimeric product, isotridachiapyrone B, has also been isolated by Schmitz.

## Results and Discussion

**Scheme 2:**
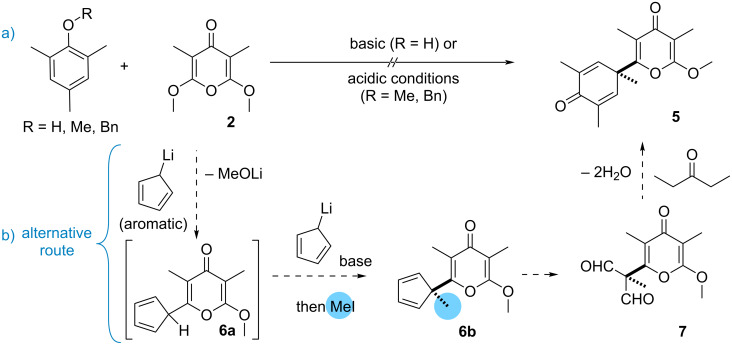
Desymmetrization of **2**.

At first glance, the structure of 2,5-cyclohexadienone **5** suggests a disconnection involving the dearomative addition of 2,4,6-trimethylphenol to α,α’-dimethoxy-γ-pyrone **2** that we eagerly sought to establish under basic activation of the nucleophile or by protonation of **2** (Scheme 2a). This ambitious coupling, however, met a dead-end and a less direct approach was explored. With a more reactive and less hindered nucleophile, we explored the coupling of lithiocyclopentadiene to compound **2**. After conjugate addition and elimination of lithium methoxide, the resulting **6a** would be deprotonated by lithiocyclopentadiene and the enolate intercepted with an alkylating reagent to build the quaternary carbon of **6b** (Scheme 2b). This one-pot procedure was reminiscent of our previous study describing the addition of an allylic carbanion, generated from allylstannane with *n*-BuLi, to **2** which was followed by the addition of an aldehyde resulting in a regioselective aldolization [29]. Thereafter, we hypothesized converting cyclopentadiene **6b** into 2,5-cyclohexadienone **5** by a sequence involving the oxidation into dialdehyde **7** and treatment with pentan-3-one to enable sequential steps of aldolization and crotonization. As an aromatic carbanion, it was unclear whether the addition of lithiocyclopentadiene to **2** would succeed [30].

**Scheme 3:**
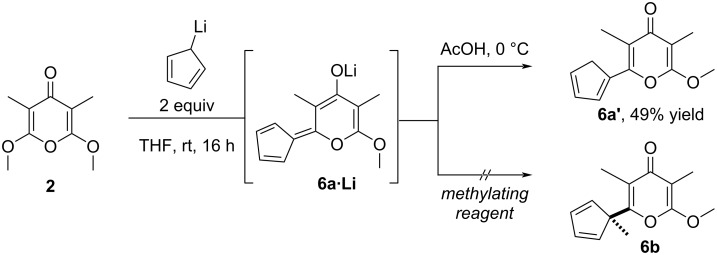
Addition of lithiocyclopentadiene to pyrone **2**.

Pleasingly though, the coupling was successful and moreover simply implemented by reacting lithiocyclopentadiene (2 equiv) with pyrone **2** at room temperature, no reaction occurring at lower temperature in contrast with the nucleophile 2-lithio-1,3-dithiane, and with acetic acid as electrophile (Scheme 3). Among the possible isomers that can be expected, a single one **6a’** was isolated in 49% yield after trituration, as it was found rather unstable on silica gel. While the addition of more reactive carbanions of 1,3-dithiane [27], allyl [27], and methyldienylbenzene groups [31] to compound **2** were demonstrated, this result amounts to the first grafting of an aromatic nucleophile to the motif.

The construction of the vicinal quaternary carbon of **6b** was next investigated by methylation of the highly delocalized enolate intermediate **6a·Li**. To that end, the addition of lithiocyclopentadiene to **2** was followed by the attendant quenching with a methylating reagent (MeI, Me_2_SO_4_, MeOTf) but a complex mixture of products was consistently obtained. To gather information on the reactivity of **6a·Li**, the stabilized enolate was treated with 4-nitrobenzaldehyde to promote the aldolization reaction but the corresponding alcohol was not observed, which confirmed the reluctance of **6a·Li** to react with other electrophiles than protons.

**Scheme 4:**
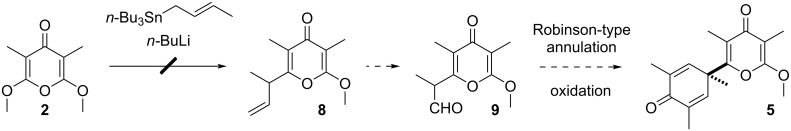
Plan to reach 2,5-cyclohexadienone **5**.

Unable to form the quaternary carbon of the target from this intermediate, the pathway to 2,5-cyclohexanedione **5** was accordingly updated and an approach to make use of the Robinson-type annulation was devised from aldehyde **9**, prepared by oxidation of α-crotyl-α’-methoxy-γ-pyrone **8** (Scheme 4). While its synthesis was initially investigated by the coupling of tri(*n*-butyl)crotylstannane to **2** in the presence of *n*-BuLi, the direct transfer of the crotyl appendage failed, likely compromised by the steric hindrance of the nucleophile [31].

**Scheme 5:**
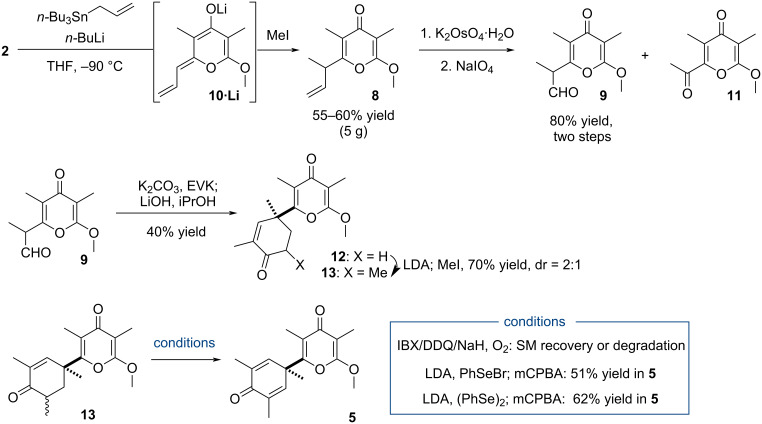
Preparation of 2,5-cyclohexadienone **5**.

To alleviate this shortcoming, the crotyl appendage of **8** was assembled by methylation of the allylic pyrone counterpart (Scheme 5). Implemented by exposing **2** to the combination of reagents tri(*n*-butyl)allylstannane/*n*-BuLi (2 equiv) followed by the treatment with MeI, the crotyl derivative **8** was directly isolated in 55–60% yield (5 g scale) via the regioselective methylation of the intermediate vinylogous enolate **10·Li**. The preparation of aldehyde **9** was carried out through a two-step sequence including dihydroxylation (K_2_OsO_4_·H_2_O, 90% yield) of **8** and oxidative cleavage (NaIO_4_, 91% yield) of the diol intermediate. Note that both ozonolysis and the one-pot Lemieux–Johnson oxidative cleavage process of **8** led instead to methyl ketone **11** in a significant amount (ca. 75% yield), probably by oxidation of the enol form of **9** [32]. The sensitive aldehyde was thus used without purification to investigate the Robinson-type annulation and a protocol was identified allowing the preparation of cyclohexenone **12** in 40% yield (2-step). Accordingly, the Michael addition of ethyl vinyl ketone (EVK), promoted by K_2_CO_3_ in a biphasic media (PhMe/H_2_O), was followed by basic treatment (LiOH) of the keto aldehyde. Since compound **12** bears the desired quaternary carbon of this family of natural products, it was pleasing to reach this milestone, keeping in mind that the fair yield of this two-step transformation may be the consequence of the aldehyde’s low stability.

Note that the Kotsuki method to perform the Robinson-type annulation of **9** with EVK, catalyzed by a combination of 1,2-cyclohexanediamine and 1,2-cyclohexanedicarboxylic acid, led to product **12** with higher yield but the scale-up of the reaction was less efficient [33]. It has to be underlined that all attempts, using either methods, to couple aldehyde **9** with 2-methylpent-1-en-3-one and directly produce **13** were unsuccessful. To achieve this goal a methylation step of **12** (LiN(iPr)_2_ (LDA); MeI) was thus necessary (70% yield, 2:1 dr).

The desaturation of the enone compound was next examined and while exposure of **13** to oxidant (*o*-iodoxybenzoic acid (IBX) or 2,3-dichloro-5,6-dicyano-1,4-benzoquinone (DDQ)) left the starting materials unchanged, treatment with NaH in the presence of oxygen to induce the aerobic oxidation caused instead the degradation of **13**. An indirect approach was more rewarding as treatment of the enolate of **13** with PhSeBr led to 2,5-cyclohexadienone **5** in 51% yield after oxidation of the selenoether intermediate with *m*-chloroperoxybenzoic acid (*m*-CPBA). An appreciable increase of the yield was actually noted with (PhSe)_2_ as electrophile, **5** being obtained in 62% yield, enabling thus an evaluation of the next desymmetrization step.

**Scheme 6:**
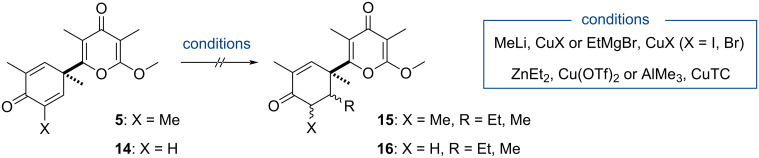
Attempts to perform the conjugate addition.

An overview of the scientific literature revealed that, while the asymmetric desymmetrization of prochiral 2,5-cyclohexadienones is a rich topic of investigation, it is mostly restricted to substrates bearing a tertiary alkoxy group [34]. Few examples of this intermolecular strategy actually involve substrates containing a quaternary carbon and the formation of a C–C bond [35–36]. Of note was the report of Takemoto and Iwata describing the 1,4-addition of AlMe_3_ to 4,4-dimethyl-2,5-cyclohexadienone in the presence of a copper salt/chiral ligand and silylating reagent [37–38]. The racemic conjugate addition of nucleophiles to **5** was first investigated, starting with the Gilman reagent which was used in Takemoto and Iwata study (Scheme 6). In addition, a screening of various organocopper reagents (prepared from MeLi, EtMgBr, ZnEt_2_ or AlMe_3_ and copper halide or thiophene-2-carboxylate (CuTC)) was conducted, to no avail. In most cases, the starting material was recovered without indication that the pyrone ring interacted instead with the reagent. To decrease the steric hindrance of the electrophile and potentially facilitate the 1,4-addition, 2,5-cyclohexadienone **14**, deprived of a methyl group, was prepared and tested but the reactivity was not significantly improved compared to **5**. Both compounds appeared thus mostly reluctant to 1,4-addition and, when it was not the case, the process was poorly reproducible.

**Scheme 7:**
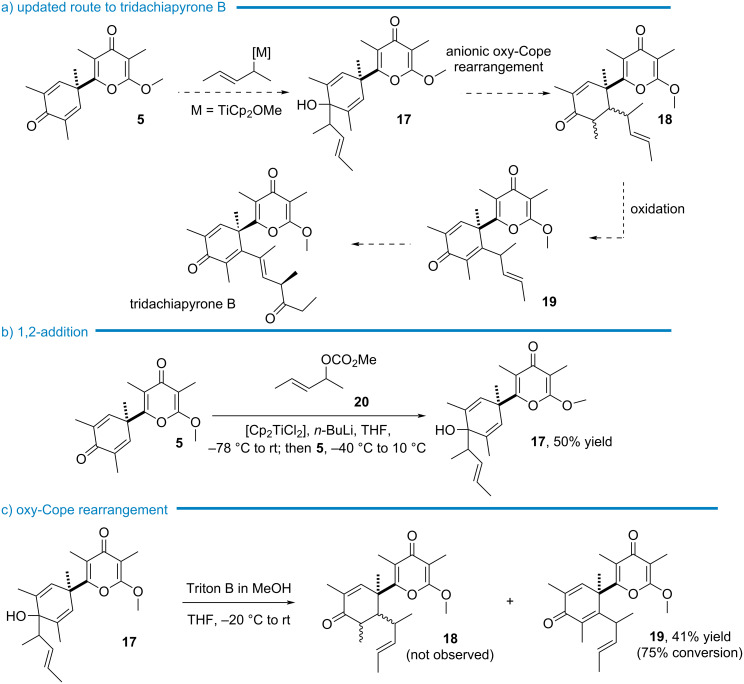
Updated route to tridachiapyrone B.

On the other hand, the 1,2-addition of Grignard reagents to **5** was observed, providing thus an alternative way of grafting a side chain. As summarized in Scheme 7a, this was envisaged through a sequence encompassing the 1,2-addition of the 1,3-dimethylallyl motif to **5** giving **17**, followed by the anionic oxy-Cope rearrangement of the dienol into cyclohexenone **18**. After desaturation, the resulting 2,5-cyclohexadienone **19** would provide a modular platform to construct the side chain of the target and analogues. Note that this updated route required the 1,2-addition of a rather hindered nucleophile to a carbonyl electrophile with low reactivity. Precedents were noted though, as Carreira used this strategy for the synthesis of indoxamycine B exploiting the reactivity of 1,3-dimethylallyltitanocene species [39]. The 1,2-addition of various allyltitanocene reagents to carbonyl compounds was described by Sato which were prepared from the corresponding allyl carbonates exposed to the combination of [Cp_2_TiCl_2_]/*n*-BuLi [40]. Pleasingly, conducting the coupling of carbonate **20** to **5** in conditions inspired from Carreira’s study (Scheme 7b) led to the desired 1,2-adduct **17** in 50% yield [41].

To perform the anionic oxy-Cope rearrangement, alcohol **17** was exposed to *t*-BuOK, in the presence of 18-crown-6 ether (−78 °C to rt) [42].

However, these conditions did not trigger the rearrangement and the starting material was recovered. On the other hand, treatment with KH in DMSO at room temperature caused the degradation of **17**. Scarcely examined for this purpose, hydroxide of quaternary ammonium salt was next evaluated to promote the anionic oxy-Cope rearrangement with the prospect that non-coordinating organic cations could facilitate the transformation by destabilizing the negative charge of the anion (Scheme 7c). Simply generated with Triton B (benzyltrimethylammonium hydroxide), the alcoholate of **17** smoothly (−20 °C to rt) underwent the [3,3]-sigmatropic rearrangement (75% conversion) directly affording 2,5-cyclohexadienone *E*-**19** which was isolated in 41% yield [43] while cyclohexenone **18** was not observed in the crude reaction mixture (as analyzed by ^1^H NMR spectroscopy). Even though the mechanism was not investigated, the presence of oxygen during the rearrangement step was suspected to account for the oxidation of the enolate intermediate, enabling thus a practical one-pot preparation of 2,5-cyclohexadienone **19** from **17**.

## Conclusion

While the construction of the side chain’s target remains to be completed, we developed a convergent access to a highly substituted 2,5-cyclohexadienone motif bearing a quaternary carbon connected to α’-methoxy-γ-pyrone, simplified analogues of tridachiapyrone B such as **19**. Without requiring the construction of polyenes, the route is enabled by key transformations such as the formal crotylation of α,α’-dimethoxy-γ-pyrone, the Robinson-type annulation of a sensitive aldehyde and 1,2-addition to 2,5-cyclohexadienone followed by an oxidative anionic oxy-Cope rearrangement promoted by hydroxide of quaternary ammonium salt. Moreover, the coupling of lithiocyclopentadiene to α,α’-dimethoxy-γ-pyrone was demonstrated, enlarging the scope of nucleophiles grafted to the pharmaceutically relevant α’-methoxy-γ-pyrone motif. Drawing from this work, future studies will be focused on an enantioselective access to the target.

## Supporting Information

File 1 Experimental details, ^1^H and ^13^C spectra of new compounds.
