# Supplementary material for: Synthetic study toward tridachiapyrone B
Source: Beilstein J Org Chem. 2022 Dec 19;18:1741–8. doi: 10.3762/bjoc.18.183 (PMC9795862; doi:10.3762/bjoc.18.183)

BEILSTEIN JOURNAL OF ORGANIC CHEMISTRY

Supporting Information

for

Synthetic study toward tridachiapyrone B

Morgan Cormier, Florian Hernvann and Michaël De Paolis

Beilstein J. Org. Chem. **2022,** *18,* 1741–1748. doi:10.3762/bjoc.18.183

Experimental details, ^{1}H and ^{13}C spectra of new compounds

License and Terms: This is a supporting information file under the terms of the Creative Commons Attribution License (https://creativecommons.org/licenses/by/4.0). Please note that the reuse, redistribution and reproduction in particular requires that the author(s) and source are credited and that individual graphics may be subject to special legal provisions.
The license is subject to the *Beilstein Journal of Organic Chemistry* terms and conditions: (https://www.beilstein-journals.org/bjoc/terms)

Table of contents

1.General information S2
1.Experimental part S2
3.NMR spectra S12

1. General Information

Dry tetrahydrofuran (THF) and methylene chloride (CH_2Cl_2) were obtained by distillation (from CaH_2 for CH_2Cl_2, sodium/benzophenone for THF). Isopropanol (iPrOH) and *N*,*N*'-dimethylformamide (DMF) were purchased in anhydrous form and used without further purification. Reagents were purchased at the highest commercial quality and used without further purification, unless otherwise stated. Reactions were monitored by thin layer chromatography (TLC) carried out on Merck silica gel plates with QF-254 indicator and an ethanolic solution of ammonium molybdate or potassium permanganate and heat as developing agents. Merck silica gel (60, particle size 0.040–0.063 mm) was used for flash column chromatography. NMR spectra were recorded on a Bruker Avance DMX 300 MHz instrument at 293 K and calibrated using residual undeuterated solvent as an internal reference (7.26 ppm and 77.00 ppm for ^{1}H and ^{13}C NMR in $CDCl_3$). The following abbreviations were used to describe the multiplicities: s = singlet, d = doublet, t = triplet, q = quartet, quint = quintet, m = multiplet, pent = pentet, hex = hexet, br = broad. IR spectra were recorded on a PerkinElmer Spectrum 100 FT-IR-spectrometer with only major peaks being reported. High resolution mass spectra (HRMS) were recorded on an LCT Premier XE benchtop orthogonal acceleration time-of-flight (TOF) mass spectrometer (Waters Micromass).

2. Experimental part

2-(Cyclopenta-1,3-dien-1-yl)-6-methoxy-3,5-dimethyl-4*H*-pyran-4-one (**6a’**)

A solution of freshly distilled cyclopentadiene (71.8 mg, 1.1 mmol, 2 equiv), that was stored at −78 °C, in anhydrous THF (5 mL) was cooled to −78 °C under argon atmosphere in a 50 mL single-necked round-bottomed flask. *n*-BuLi (0.75 mL, 1.2 mmol, 1.2 equiv, *c* = 1.6 M in hexane) was added at −78 °C and stirred at this temperature for 45 min. Solubilized in 5 mL of THF, α,α′-dimethoxy-γ-pyrone **2**

(101 mg, 0.55 mmol, 1 equiv) was introduced (over 10 min) at −78 °C. The cooling bath was removed and the solution was stirred overnight at room temperature, before being cooled to 0 °C and treated with AcOH (0.1 mL, 1.6 mmol, 3 equiv). The combined organic layers were washed with brine and $NaHCO_3$ (saturated aqueous solution), dried ($MgSO_4$), filtered, and concentrated under reduced pressure. The residue was triturated (iPr_2O/pentane 3:7) and the resulting solution was concentrated to give **6a'** (58 mg, 49% yield), as yellow oil (caution: the product is unstable on silica gel).

^{1}H NMR (300MHz, $CDCl_3$): δ [ppm] = 7.04–7.02 (m, 1H), 6.66-6.61 (m, 2H), 3.98 (s, 3H), 3.43–3.40 (m, 2H), 2.13 (s, 3H), 1.83 (s, 3H). **^{13}C NMR** (75MHz, $CDCl_3$): δ [ppm] = 181.2, 161.7, 152.1, 138.6, 136.2 (2C), 133.1, 117.6, 99.2, 55.4, 42.9, 11.2, 7.2. **IR** (neat, cm^{-1}): 3414, 2957, 1740, 1652, 1575, 1463, 1408, 1374, 1327, 1163, 1034, 966, 767, 667, 475. **HRMS** (ESI) *m/z* for $C_{13}H_{15}O_3$ $(M+H)^+$: 219.1020, calculated: 219.1021

2-(But-3-en-2-yl)-6-methoxy-3,5-dimethyl-4*H*-pyran-4-one (**8**)

A solution of **2** (100 mg, 0.54 mmol, 1 equiv) and tri(*n*-butyl) allylstannane (360 mg, 337 µL, 1.08 mmol, 2 equiv) in THF (5 mL, *c* = 0.1 M) was brought to −90 °C. After the dropwise introduction of *n*-BuLi (0.75 mL, 2.2 equiv, *c* = 1.6 M in hexane), the color of the solution turned from colorless to bright red. After 30 min at this temperature, MeI (230 mg, 1.3 mmol, 3 equiv) was added, the mixture was stirred between −90 °C and −78 °C for 30 min, then the cooling bath was removed to let the flask reach room temperature. After 1 h, an aqueous saturated solution of NH_4Cl (10 mL) was added at 0 °C, and the mixture was extracted with CH_2Cl_2 (3 × 10 mL), washed with brine, and dried with Na_2SO_4. After filtration and evaporation of the volatiles, the residue was purified by flash chromatography on silica gel (cyclohexane/AcOEt 6:4) to give **8** (73 mg, 60% yield), as yellow oil (R_f = 0.2 cyclohexane/AcOEt 6:4).

5 g scale experiment: A solution of **2** (5.0 g, 27.2 mmol, 1 equiv) and tri(*n*-butyl) allylstannane (18.0 g, 16.9 mL, 54.3 mmol, 2 equiv) in THF (200 mL, *c* = 0.14 M) was brought to −90 °C. After the dropwise introduction of *n*-BuLi (37 mL, 59.8 mmol, 2.2 equiv, *c* = 1.6 M in hexane), the color of the solution turned from colorless to bright red. After 30 min at this temperature, MeI (11.6 g, 5.1 mL, 81.6 mmol, 3 equiv) was added, the mixture was stirred between −90 °C and −78 °C for 30 min, then the cooling bath was removed to let the flask reach the room temperature. After 1 h, an aqueous saturated solution of NH_4Cl (200 mL) was added at 0 °C, and the mixture was extracted with CH_2Cl_2 (3 × 150 mL), washed with brine, and dried with Na_2SO_4. After filtration and evaporation of the volatiles, the residue was purified by flash chromatography on silica gel (cyclohexane/AcOEt 6:4) to give **8** (3.1 g, 55% yield).

^{1}H NMR (300 MHz, $CDCl_3$): *δ* [ppm] = 5.90 (ddd, *J* = 16.9, 10.5, 6.4 Hz, 1H), 5.14–5.07 (m, 2H), 3.94 (s, 3H), 3.71–3.66 (m, 1H), 1.96 (s, 3H), 1.83 (s, 3H), 1.35 (d, *J* = 7.0 Hz, 3H). **^{13}C NMR** (75 MHz, $CDCl_3$): *δ* [ppm] = 181.2, 162.2, 158.9, 137.7, 117.9, 115.7, 99.4, 55.3, 38.8, 17.1, 9.6, 6.9. **IR** (neat, cm^{-1}): 2929, 1663, 1594, 1458, 1406, 1376, 1319, 1247, 1166, 1033, 981, 918, 799, 769, 724, 671, 474. **HRMS** (ESI) *m/z* for $C_{12}H_{17}O_3$ $(M+H)^+$: 209.1101, calculated: 209.1103

2-(6-Methoxy-3,5-dimethyl-4-oxo-4*H*-pyran-2-yl)propanal (**9**)

2-(But-3-en-2-yl)-6-methoxy-3,5-dimethyl-4*H*-pyran-4-one (**8**, 590 mg, 2.83 mmol, 1 equiv) was dissolved in a mixture of MeCN/H_2O (30 mL/1.5 mL, 95:5). $K_2OsO_4 \cdot H_2O$ (52 mg, 0.14 mmol, 0.05 equiv) and *N*-methylmorpholine oxide (664 mg, 5.67 mmol, 2 equiv) were added. The solution was stirred overnight at room temperature and the volatiles were removed under reduced pressure. The residue was purified by flash

chromatography on silica gel (AcOEt/CH_3OH 100:0→95:5) to give the diol intermediate (613 mg, 2.53 mmol, 90%), as colorless oil. The material (613 mg, 2.53 mmol, 1 equiv) was dissolved in a mixture of MeCN/H_2O (24 mL/6 mL, 4:1). $NaIO_4$ (813 mg, 3.8 mmol, 1.5 equiv) was added and the reaction was stirred at room temperature for 1 h before addition of an aqueous solution of HCl (1 M) and AcOEt. The two layers were separated, the aqueous layer was extracted with AcOEt and the combined organic layers were washed with brine, dried over $MgSO_4$ and concentrated. The residue (484 mg, 2.3 mmol, 91%) was used without further purification due to its sensitivity.

^{1}H NMR (300MHz, $CDCl_3$): δ [ppm] = 9.72 (s, 1H), 3.91 (s, 3H) 3.89–3.81 (m, 1H), 2.00 (s, 3H), 1.85 (s, 3H), 1.47 (d, J = 7.2 Hz, 3H).

2-Acetyl-6-methoxy-3,5-dimethyl-4H-pyran-4-one (**11**)[11]

R_f = 0.15 (Cyclohexane/AcOEt, 7:3): **^{1}H NMR** (300MHz, $CDCl_3$): δ [ppm] = 4.07 (s, 3H), 2.53 (s, 3H), 2.31 (s, 3H), 1.90 (s, 3H).**^{13}C NMR** (75MHz, $CDCl_3$): δ [ppm] = 193.5, 180.4, 161.5, 148.7, 126.6, 102.0, 55.8, 28.1, 10.2, 7.3. **MS** (ESI) *m/z*: 197.08 $(M+H)^+$

2-(1,3-Dimethyl-4-oxocyclohex-2-en-1-yl)-6-methoxy-3,5-dimethyl-4*H*-pyran-4-one (**12**)

To a solution of crude aldehyde **9** (484 mg, 2.3 mmol) in toluene (20 mL), was added an aqueous saturated solution of K_2CO_3 (6 mL) followed by EVK (0.6 mL, 6.9 mmol, 3 equiv). The biphasic mixture was stirred at room temperature overnight, aqueous NH_4Cl saturated solution and AcOEt were introduced, and the two layers were separated. The aqueous layer was extracted with AcOEt and the combined organic layers were washed with brine, dried over $MgSO_4$ and concentrated under reduced pressure.

The residue was dissolved in 2-propanol (20 mL) and $LiOH\cdot H_2O$ (28 mg, 1.15 mmol, 0.5 equiv) was added in one portion. The solution was stirred at room temperature overnight before the addition of aqueous saturated solution of NH_4Cl and AcOEt. The two layers were separated and the aqueous layer was extracted with AcOEt. The combined organic layers were washed with brine, dried over $MgSO_4$ and concentrated. The residue was purified by flash chromatography on silica gel (cyclohexane/AcOEt) to give **12** (250 mg, 0.92 mmol, 40%), as yellow oil, R_f = 0.36 (cyclohexane/AcOEt 1:1). **^{1}H NMR** (300 MHz, $CDCl_3$): δ [ppm] = 6.67–6.65 (m, 1H), 3.88 (s, 3H), 2.54–2.25 (m, 4H), 1.93 (s, 3H), 1.76 (d, J = 1.4 Hz, 3H), 1.75 (s, 3H), 1.53 (s, 3H). **^{13}C NMR** (75 MHz, $CDCl_3$): δ [ppm] = 198.0, 181.3, 161.8, 159.6, 149.4, 134.3, 119.7, 99.4, 55.5, 42.8, 34.7, 34.6, 24.7, 16.1, 10.8, 7.0. **IR** (neat, cm^{-1}): 2925, 2854, 1711, 1654, 1576, 1463, 1407, 1319, 1252, 1165, 1018, 981, 801, 769, 671, 474. **HRMS** (ESI) *m/z* for $C_{16}H_{21}O_4$ $(M+H)^+$: 277.1422, calculated: 277.1420

2-Methoxy-3,5-dimethyl-6-(1,3,5-trimethyl-4-oxocyclohex-2-en-1-yl)-4*H*-pyran-4-one (**13**)

To a solution of freshly prepared LDA (11.8 mL, 3.3 mmol, 1.3 equiv, c = 0.28 M in anhydrous THF) under argon was added enone **12** (700 mg, 2.5 mmol, 1 equiv) in anhydrous THF (10 mL) at −78 °C over 10 min. After the addition, the mixture was stirred for 1 h at this temperature, the solution becoming progressively orange. MeI (0.79 mL, 12.7 mmol, 5 equiv) was added at −78°C and after 30 min at this temperature the cooling bath was removed, the flask reaching room temperature. After 1 h, the orange and cloudy solution was treated with aqueous saturated solution of NH_4Cl and extracted with Et_2O. The combined organic layers were washed with brine, dried with Na_2SO_4, filtered, and concentrated under reduced pressure. The residue was purified

by flash chromatography on silica gel (cyclohexane/AcOEt 7:3) to give **13** (507 mg, 70% yield, dr = 2:1), a yellow oil, R_f = 0.40 (cyclohexane/AcOEt 6:4).

^{1}H NMR for 2 diastereoisomers (2*:1*) (300 MHz, $CDCl_3$): δ [ppm] = 6.65–6.65 (m, 1H), *3.98 (s, 0.9H)*, 3.91 (s, 2H), *2.72–2.63 (m, 0.3H),* 2.58−2.52 (m, 0.9H), 2.38–2.26 (m, 0.9H), *2.20–2.16 (m, 0.3H),* 2.06 (s, 2H), *1.98 (s, 0.9H),* 1.86–1.83 (m, 6H), *1.66 (s, 0.9H),* 1.52 (s, 2H), *1.17 (d, J = 6.6 Hz, 0.9H),* 1.12 (d, *J* = 6.7 Hz, 2H). **^{13}C NMR** *for major diastereoisomer* (75 MHz, $CDCl_3$): δ [ppm] = 201.1, 181.4, 161.8, 158.7, 147.7, 134.5, 119.9, 99.2, 55.5, 43.5, 43.4, 38.7, 28.1, 16.2, 15.2, 10.9,7.0. **IR** (neat, cm^{-1}): 2927, 2855, 1683, 1456, 1364, 1560, 1187, 1065, 1017, 915, 803, 491. **HRMS** (ESI) *m/z* for $C_{17}H_{23}O_4$ $(M+H)^+$: 291.1547, calculated: 291.1544

2-Methoxy-3,5-dimethyl-6-(1,3,5-trimethyl-4-oxocyclohexa-2,5-dien-1-yl)-4*H*-pyran-4-one (**5**)

To a solution of freshly prepared LDA (2.5 mL, 0.68 mmol, 2 equiv, *c* = 0.28 M in anhydrous THF) under argon was added enone **13** (100 mg, 0.34 mmol, 1 equiv) in anhydrous THF (2 mL) at −78 °C over 5 min. After 2 h at this temperature, the solution became pale orange. Diphenyl diselenide (106 mg, 0.34 mmol, 1 equiv) in 1 mL of anhydrous THF was added at −78 °C and maintained at this temperature for 1 h. Aqueous saturated solution of NH_4Cl was introduced and the biphasic system was extracted with Et_2O. The combined organic layers were washed with brine, dried (Na_2SO_4), filtered, and concentrated under reduced pressure. The residue was dissolved in CH_2Cl_2 (3 mL), and *m*-CPBA (60 mg, 0.34 mmol, 1 equiv) was added at −78 °C. The suspension was stirred at −78 °C for 30 min, the cooling bath was removed for the flask to reach room temperature over 1 h and Et_3N (47 µL, 0.34 mmol, 1 equiv) was added at this temperature. After 1 h, the reaction mixture was treated with aqueous

saturated solution of $NaHCO_3$ and extracted with CH_2Cl_2. The combined organic layers were washed with brine, dried (Na_2SO_4), filtered, and concentrated under reduced pressure. The residue was purified by flash chromatography on silica gel (cyclohexane/AcOEt 7:3) to give **5** (61 mg, 62% yield), as yellow pale oil, R_f = 0.33 (cyclohexane/AcOEt, 6:4).

^{1}H NMR (300 MHz, $CDCl_3$): δ [ppm] = 6.70 (s, 2H), 4.00 (s, 3H), 1.95 (s, 6H), 1.85 (s, 3H), 1.76 (s, 3H), 1.62 (s, 3H). **^{13}C NMR** (75 MHz, $CDCl_3$): δ [ppm] = 185.8, 180.7, 161.8, 153.7, 145.5 (2C), 134.7 (2C), 121.1, 99.8, 55.5, 45.4, 24.6, 16.0 (2C), 9.1, 6.9. **IR** (neat, cm^{-1}): 2925, 1661, 1591, 1463, 1407, 1377, 1319, 1249, 1167, 977, 769, 475. **HRMS** (ESI) *m/z* for $C_{17}H_{21}O_4$ $(M+H)^+$: 289.1445, calculated: 289.1440

2-(1,3-Dimethyl-4-oxocyclohexa-2,5-dien-1-yl)-6-méthoxy-3,5-dimethyl-4*H*-pyran-4-one (**14**)

To a solution of freshly prepared LDA (3.8 mL, 1.1 mmol, 1.5 equiv, *c* = 0.28 M in anhydrous THF) under argon was added enone **12** (200 mg, 0.72 mmol, 1 equiv) in anhydrous THF (2 mL) over 5 min at −78 °C. After the addition, the mixture was stirred for 2 h at this temperature, during which the solution became progressively pale orange. Diphenyl diselenide (225 mg, 0.72 mmol, 1 equiv) diluted in 2 mL of anhydrous THF was added at −78 °C and stirred at this temperature for 1 h, the solution turning orange-yellow. Treatment with aqueous saturated solution of NH_4Cl was followed by extraction with Et_2O. The combined organic layers were washed with brine, dried over Na_2SO_4, filtered, and concentrated under reduced pressure. The residue was dissolved in CH_2Cl_2 (5 mL) and *m*-CPBA (125 mg, 0.72 mmol, 1 equiv) was added to the mixture at −78 °C. This temperature was maintained for 30 min before removing the cooling bath for the flask to reach room temperature over 1 h. Et_3N (100 μL,

0.72 mmol, 1 equiv) was added to the mixture and, after 1 h, the reaction was treated with aqueous saturated solution of $NaHCO_3$ and extracted with CH_2Cl_2. The combined organic layers were washed with brine, dried over Na_2SO_4, filtered, and concentrated under reduced pressure. The residue was purified by flash chromatography on silica gel (cyclohexane/AcOEt 7:3) to give **14** (131 mg, 65% yield), as yellow pale oil, R_f = 0.3 (Cyclohexane/AcOEt, 6:4).

Procedure with PhSeBr:

To a solution of freshly prepared LDA (2.5 mL, 0.68 mmol, 2 equiv, *c* = 0.28 M in anhydrous THF) under argon was added enone **13** (100 mg, 0.34 mmol, 1 equiv) in anhydrous THF (2 mL) at −78 °C over 5 min. After 2 h at this temperature, the solution became pale orange. Phenylselenyl bromide (80 mg, 0.34 mmol, 1 equiv) in 1 mL of anhydrous THF was added at −78 °C and the flask was maintained at this temperature for 1 h. Aqueous saturated solution of NH_4Cl was introduced and the biphasic system was extracted with Et_2O. The combined organic layers were washed with brine, dried (Na_2SO_4), filtered, and concentrated under reduced pressure. The residue was dissolved in CH_2Cl_2 (3 mL), and *m*-CPBA (60 mg, 0.34 mmol, 1 equiv) was added at −78 °C. The suspension was stirred at −78 °C for 30 min, the cooling bath was removed for the flask to reach room temperature over 1 h and Et_3N (47 mL, 0.34 mmol, 1 equiv) was added at this temperature. After 1 h, the reaction was treated with aqueous saturated solution of $NaHCO_3$ and extracted with CH_2Cl_2. The combined organic layers were washed with brine, dried (Na_2SO_4), filtered, and concentrated under reduced pressure. The residue was purified by flash chromatography on silica gel (cyclohexane/AcOEt 7:3) to give **5** (49 mg, 51% yield).

^{1}H NMR (300 MHz, $CDCl_3$): δ [ppm] = 6.92 (dd, J = 9.8, 3.0 Hz, 1H), 6.74–6.72 (m, 1H), 6.37 (d, J = 9.9 Hz), 4.01 (s, 3H), 1.95 (d, J = 1.4 Hz, 3H), 1.85 (s, 3H), 1.79 (s, 3H), 1.65 (s, 3H). **^{13}C NMR** (75 MHz, $CDCl_3$): δ [ppm] = 185.2, 180.7, 162.0, 153.1, 150.2, 145.8, 135.4, 128.5, 121.7, 100.0, 55.7, 46.0, 24.6, 16.0, 9.3, 7.1. **IR** (neat, cm^{-1}): 2928, 1709, 1656, 1579, 1463, 1376, 1252, 1165, 980, 733, 474. **HRMS** (ESI) *m/z* for $C_{16}H_{19}O_4$ $(M+H)^+$: 275.1248, calculated: 275.1244

2-(4-Hydroxy-1,3,5-trimethyl-4-((3*E*)-pent-3-en-2-yl)cyclohexa-2,5-dien-1-yl)-6-methoxy-3,5-dimethyl-4*H*-pyran-4-one (**17**)

To a suspension of $TiCp_2Cl_2$ (52 mg, 0.21 mmol, 3 equiv) in anhydrous THF (1 mL) kept at −78 °C was added dropwise *n*-BuLi (0.26 mL, 0.42 mmol, 6 equiv, *c* = 1.6 M). The resulting mixture was stirred at −78 °C for 1 h before addition of carbonate **20** (15 mg, 0.10 mmol, 1.5 equiv), diluted in 0.5 mL of anhydrous THF. Stirring was continued for 30 min at −78 °C and then at room temperature for 20 min. The reaction mixture was cooled to −40 °C and, after 10 min, a solution of **5** (20 mg, 0.07 mmol, 1 equiv) in THF (0.5 mL) was slowly added. The reaction mixture was stirred and allowed to gradually warm up to 10 °C over 3 h. The reaction was quenched by the addition of aqueous solution of NaOH (1 M, 0.2 mL), followed by addition of Celite® (100 mg) and NaF (116 mg, 2.8 mmol, 40 equiv). Air was bubbled into the reaction mixture under vigorous stirring until the black color disappeared (ca. 20 min). The resulting yellow slurry was filtered through a plug of Celite® and the pad was washed with Et_2O (3 x 10 mL). The combined filtrate was concentrated under reduced pressure to give an orange oil that was purified by flash chromatography on silica gel (cyclohexane/AcOEt 6:4) to furnish **17** (13 mg, 50%), as yellow oil, R_f = 0.3 (cyclohexane/AcOEt 6:4).

^{1}H NMR (300 MHz, $CDCl_3$): δ [ppm] = 5.63–5.45 (m, 4H), 3.97 (s, 3H), 2.67–2.62 (m, 1H), 1.89 (s, 3H), 1.86 (d, J = 1.4 Hz, 3H), 1.84 (s, 3H), 1.79 (d, J = 1.4 Hz, 3H), 1.73 (d, J = 5.6 Hz, 3H), 1.42 (s, 3H), 0.93 (d, J = 7.1 Hz, 3H). **^{13}C NMR** (75 MHz, $CDCl_3$): δ [ppm] = 181.6, 161.7, 158.9, 135.7, 134.9, 133.3, 129.3, 129.2, 127.9, 120.6, 99.3, 74.1, 55.5, 43.5, 43.3, 25.7, 19.9, 18.3, 18.1, 16.3, 9.5, 7.1. **HRMS** (Field Desorption) *m/z* for $C_{22}H_{31}O_4$ $(M+H)^+$: 359.2227, calculated: 359.2222

2-{3,5-Dimethyl-4-oxo-2-[(3*E*)-pent-3-en-2-yl]cyclohexa-2,5-dien-1-yl}-6-methoxy-3,5-dimethyl-4*H*-pyran-4-one (**19**)

To a solution of **17** (10 mg, 0.030 mmol, 1 equiv) in distilled THF (2 mL) was added dropwise Triton B (40 μL, 0.093 mmol, 3 equiv, 40% in MeOH) at −20 °C. The cooling bath was removed, the flask was left for 4 h at room temperature before quenching with aqueous solution of citric acid 10% (0.5 mL) and extraction with Et_2O. The combined organic layers were washed with brine, dried (Na_2SO_4), filtered, and concentrated under reduced pressure. The residue was purified by preparative TLC (cyclohexane/AcOEt 7:3) to give **19** (4 mg, 41% yield), as colorless oil, R_f = 0.35 (cyclohexane/AcOEt 6:4).

^{1}H NMR (300MHz, $CDCl_3$): δ [ppm] = 6.90 (s, 1H), 5.79–5.70 (m, 1H), 5.42 (ddd, J = 15.6, 6.3, 2.1 Hz, 1H), 4.05–3.98 (m, 1H), 3.90 (s, 3H), 2.34 (s, 3H), 2.27 (s, 3H), 2.08 (s, 3H), 1.92 (s, 3H), 1.81 (s, 3H), 1.71 (ddd, J = 6.4, 1.8, 1.7 Hz, 3H), 1.41 (d, J = 7.3 Hz, 3H). **HRMS** (EI) *m/z* for $C_{22}H_{28}O_4$ $(M)^+$: 356.2023, calculated: 356.2021

3. NMR spectra

^{1}H (300 MHz, CDCl3, 293 K) of allyltributylstannane

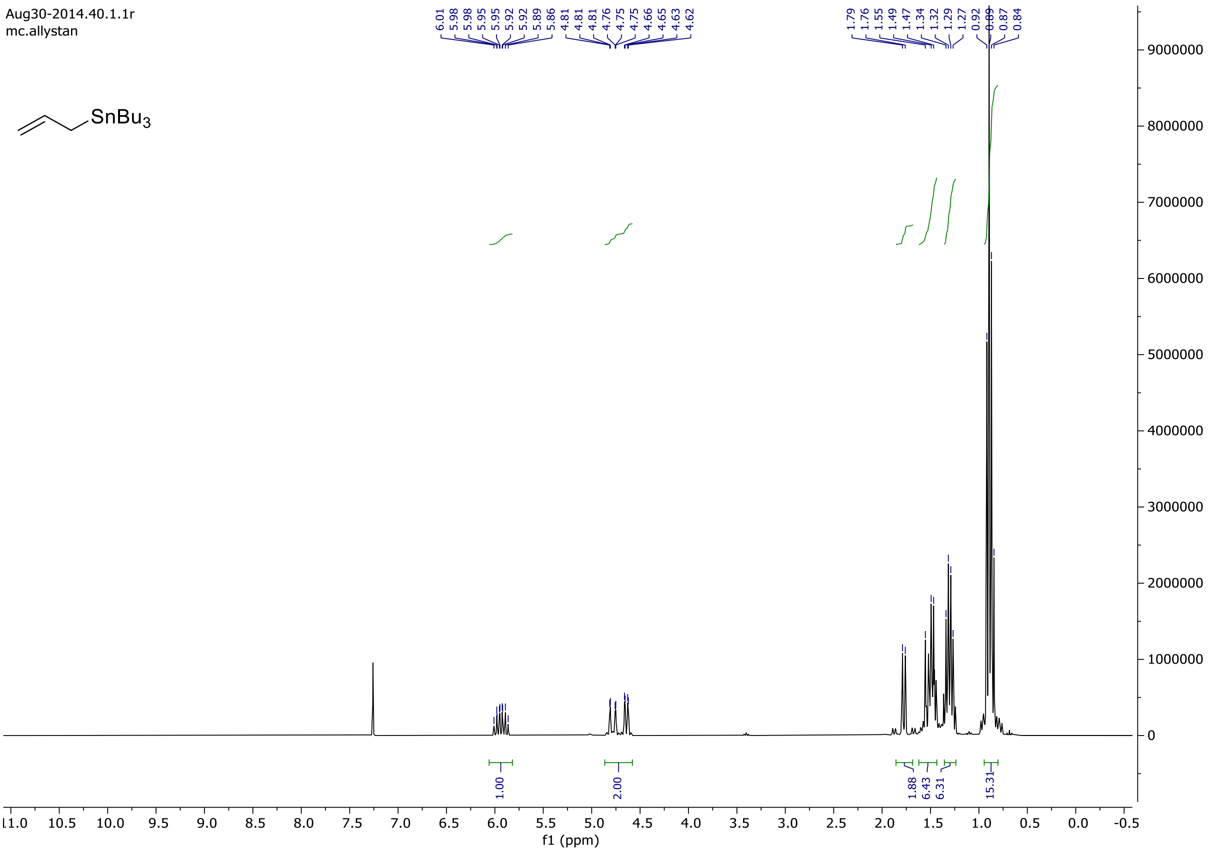

[13]C (75Mz, $CDCl_3$, 293 K) of allyltributylstannane

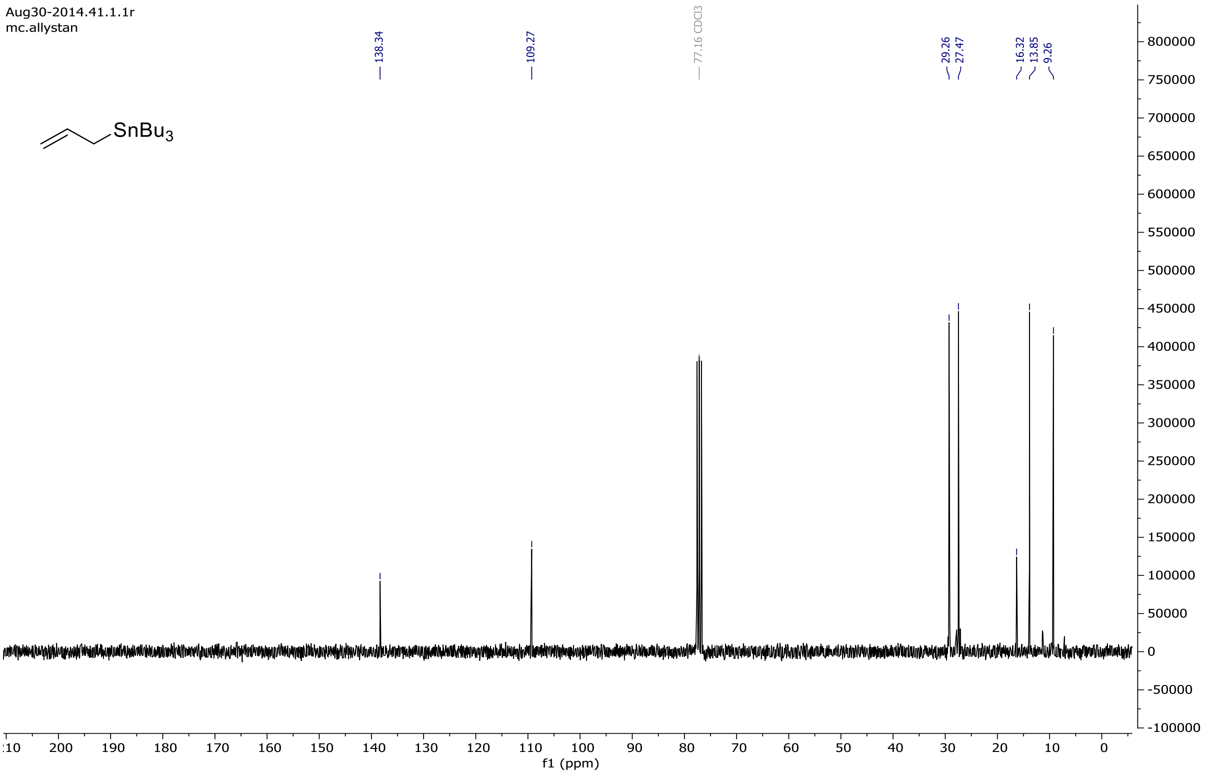

^{1}H (300 MHz, $CDCl_3$, 293 K) of crotylstannane

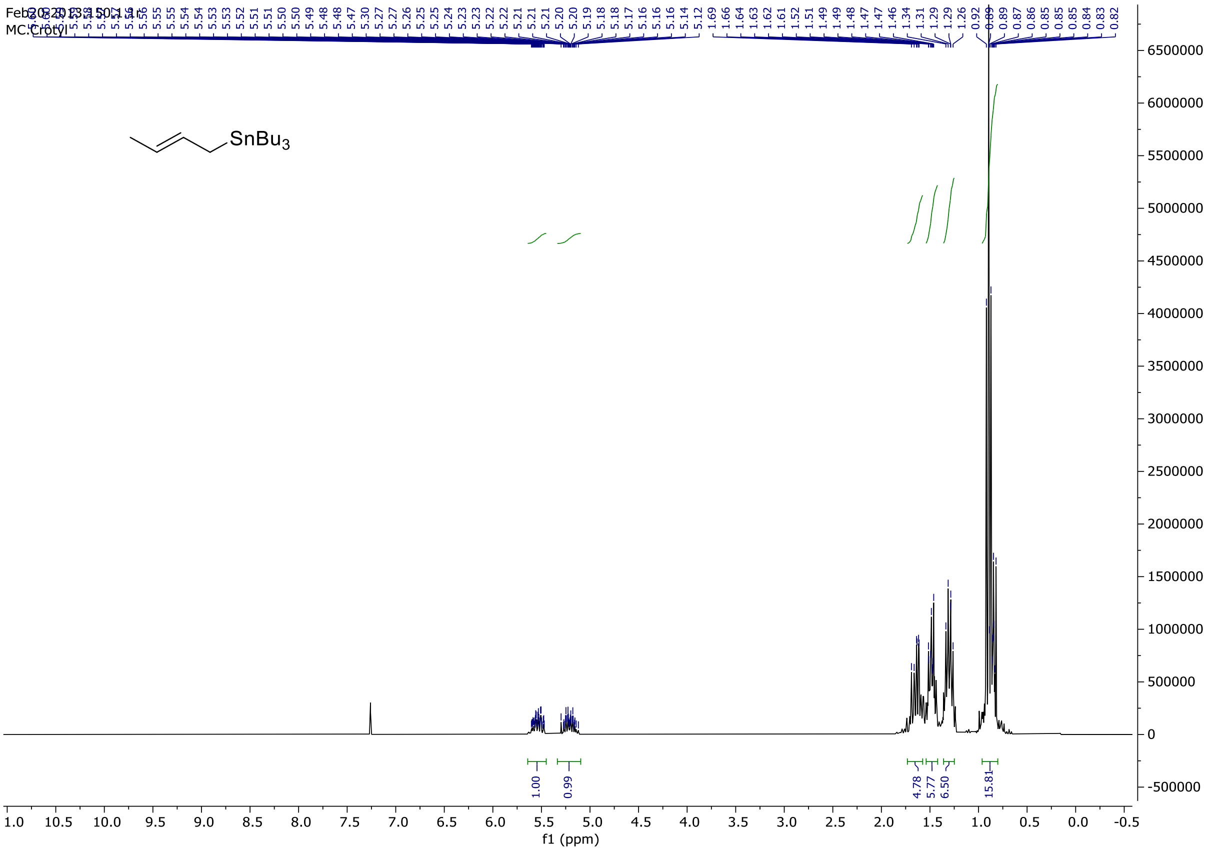

^{13}C (75Mz, $CDCl_3$, 293 K) of crotylstannane

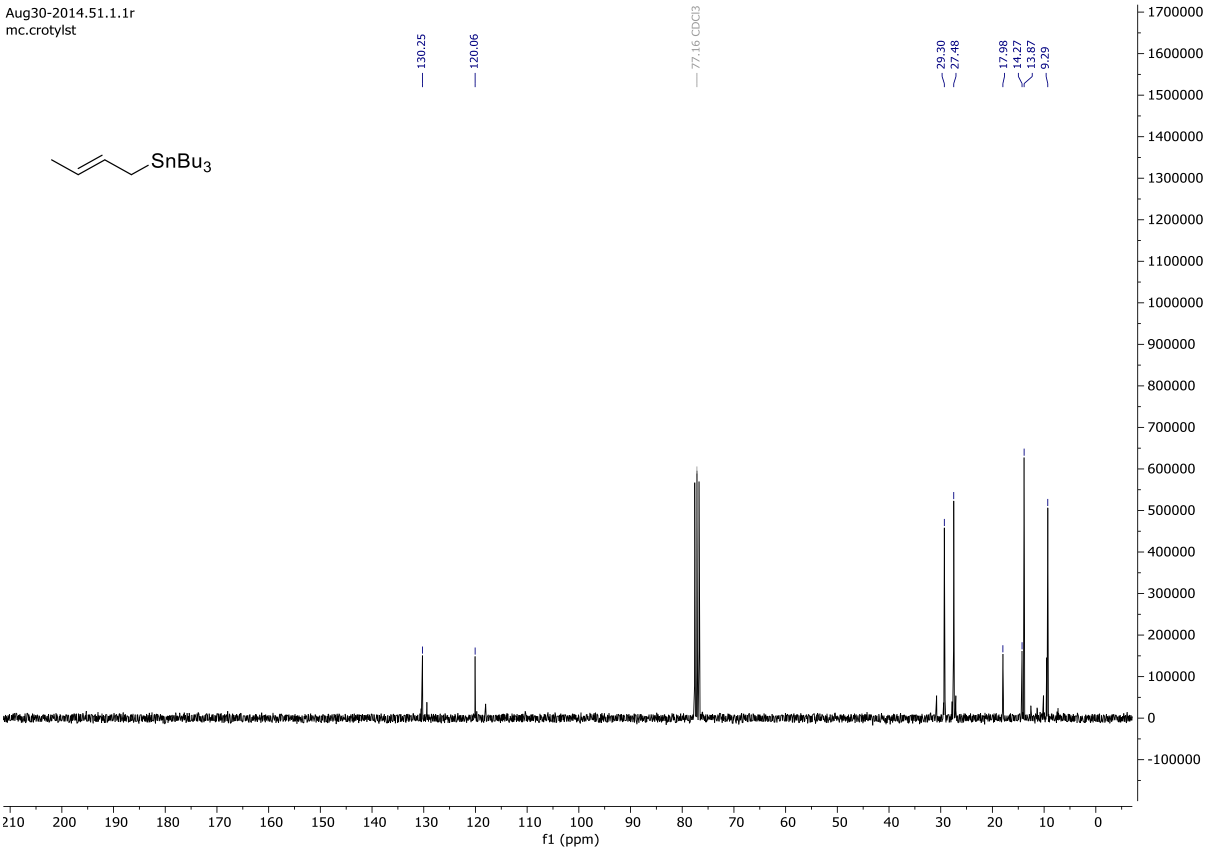

^{1}H (300 MHz, CDCl$_3$, 293 K) of (*E*)-methylpent-3-en-2-yl carbonate

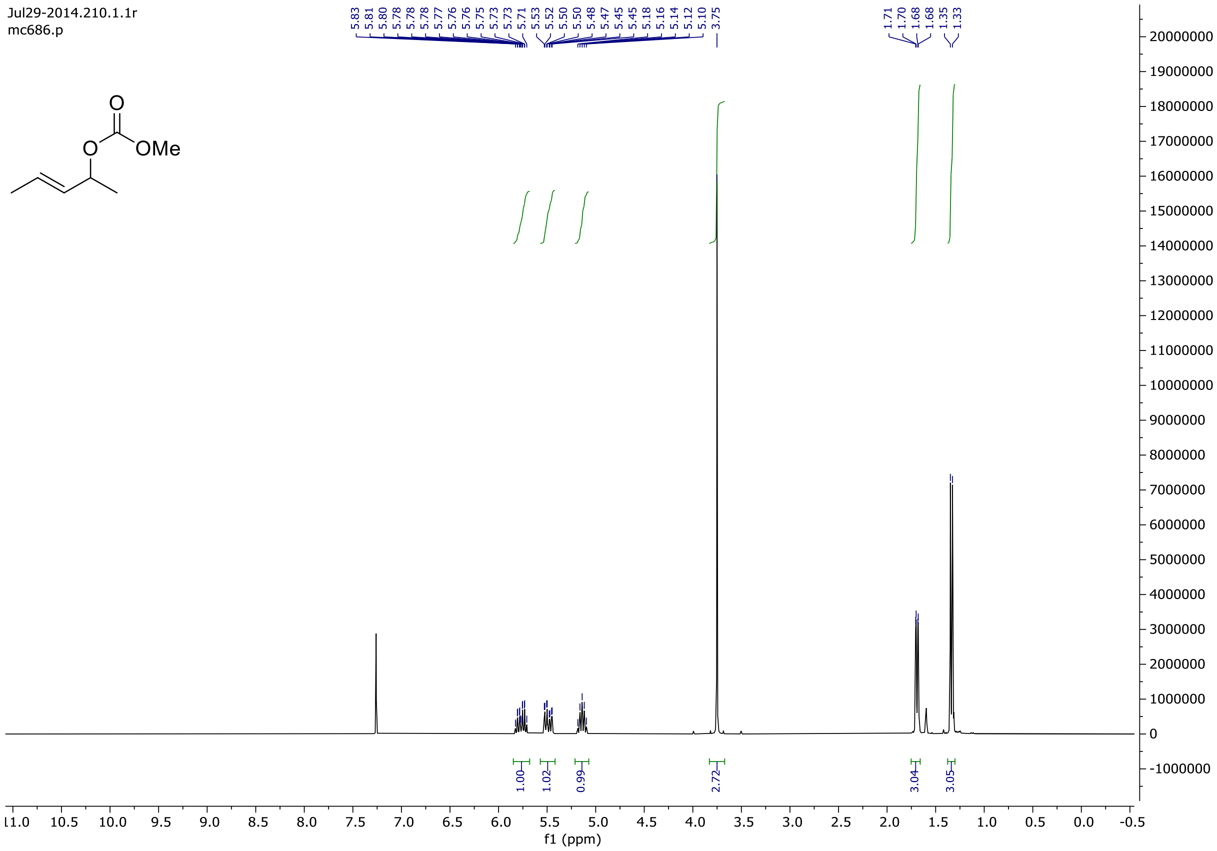

^{1}H (300 MHz, CDCl$_3$, 293 K) of α,α'-dimethoxy-γ-pyrone

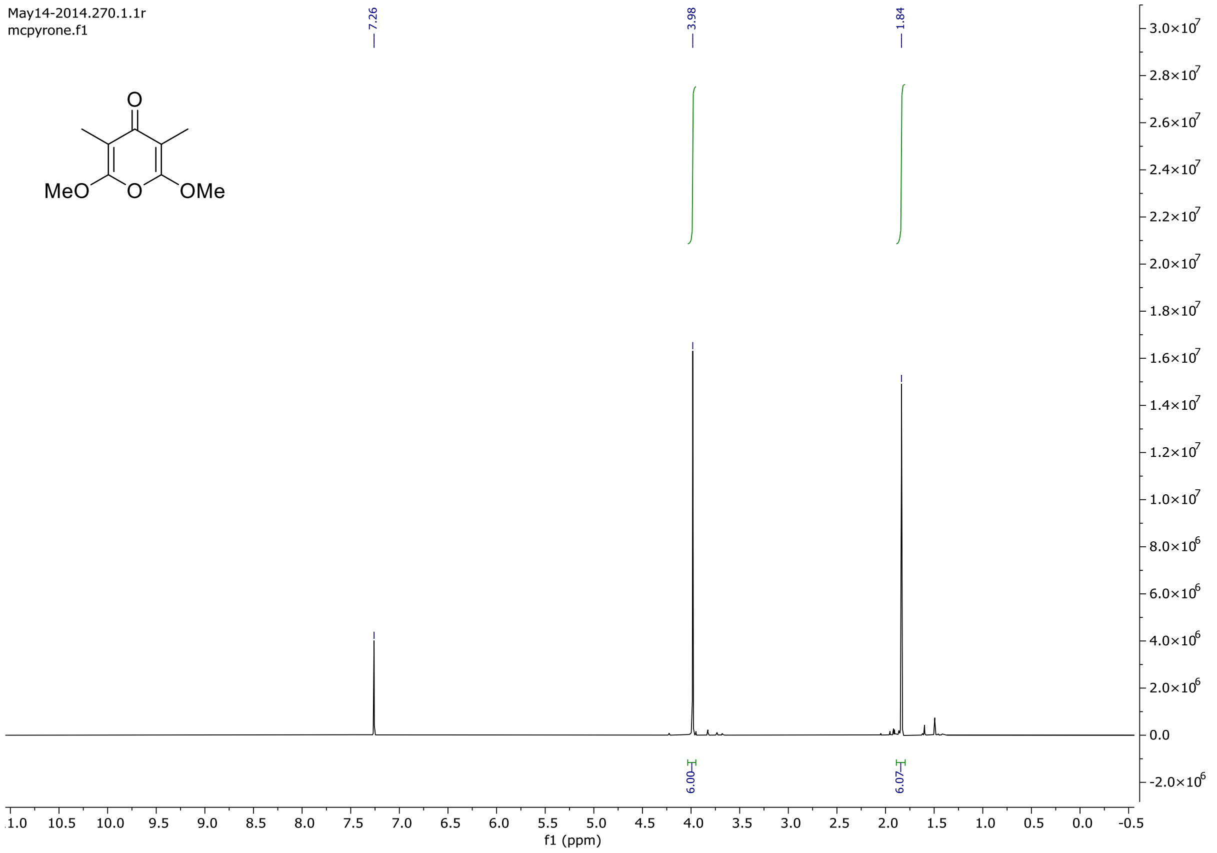

^{1}H (300 MHz, $CDCl_3$, 293 K) of 6a’

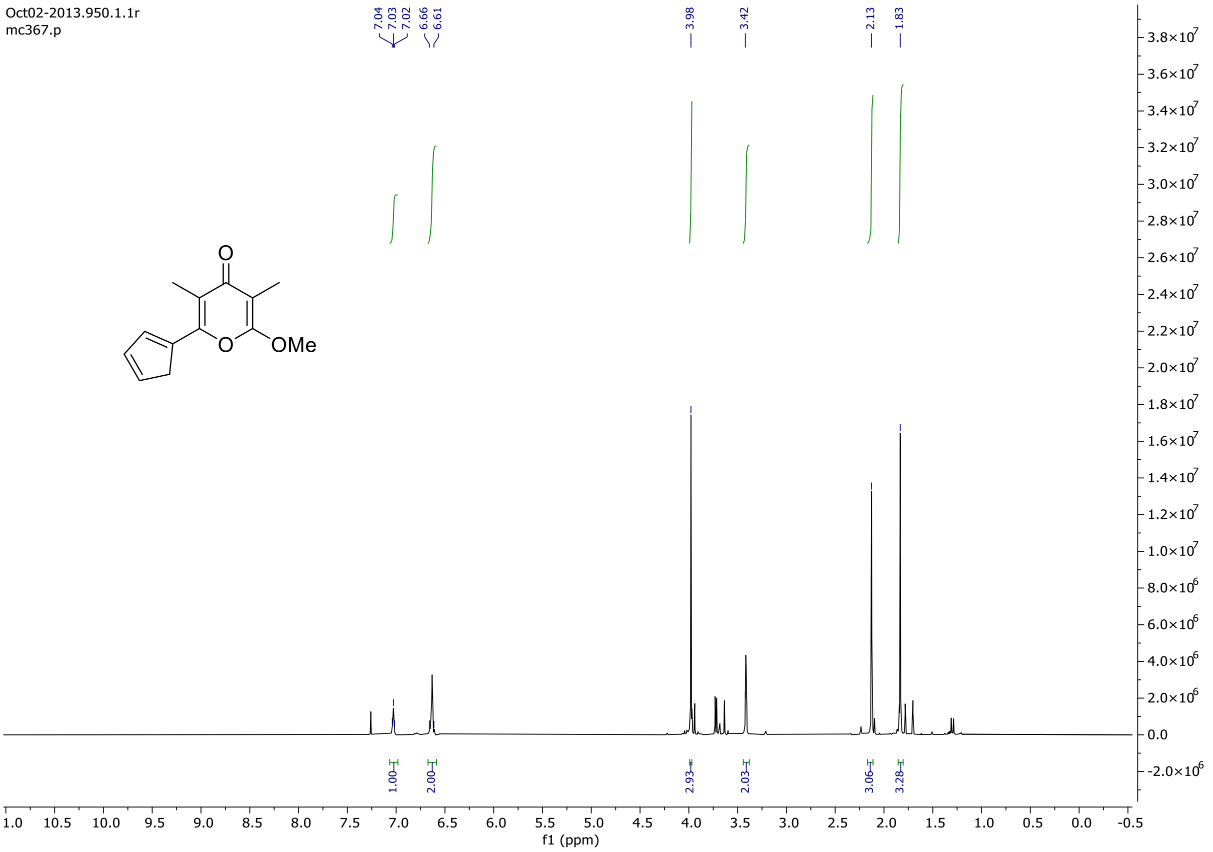

^{13}C (75Mz, $CDCl_3$, 293 K) of 6a’

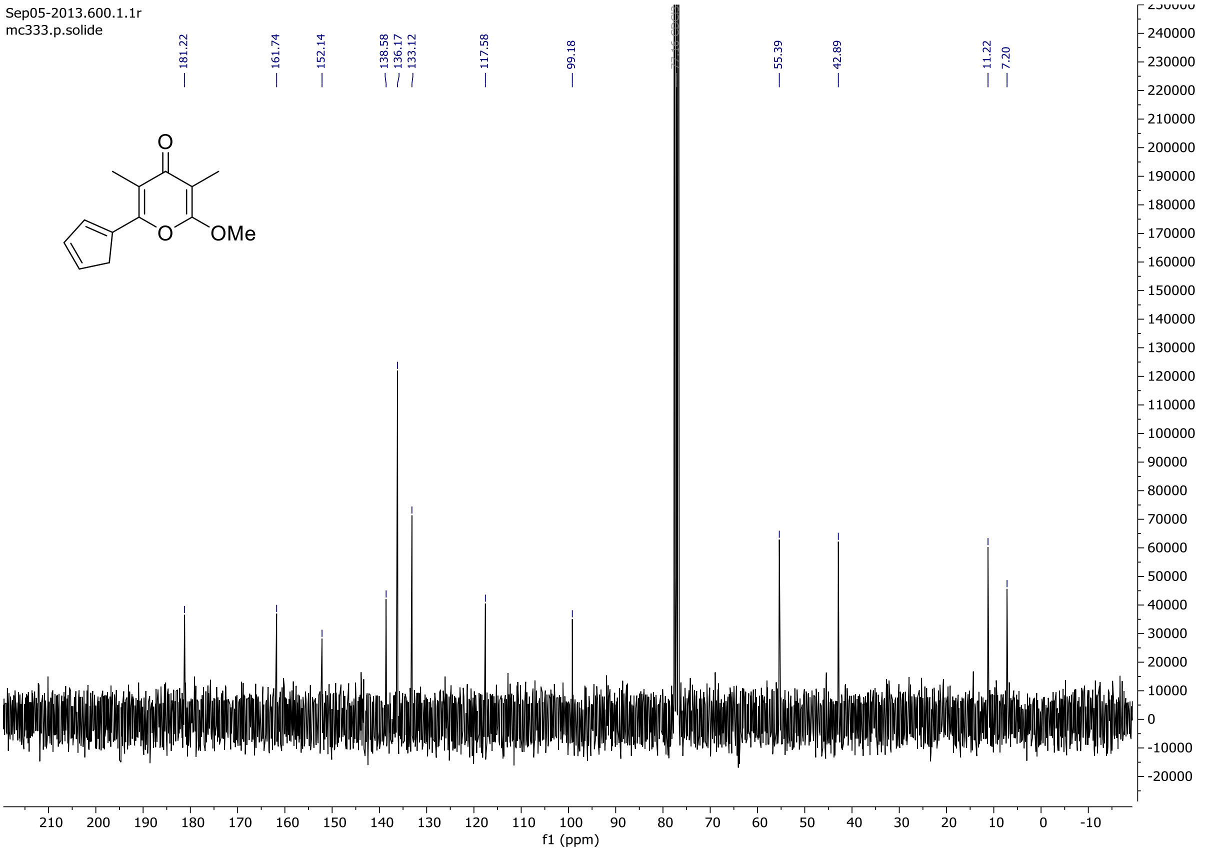

^{1}H (300 MHz, $CDCl_3$, 293 K) of 8

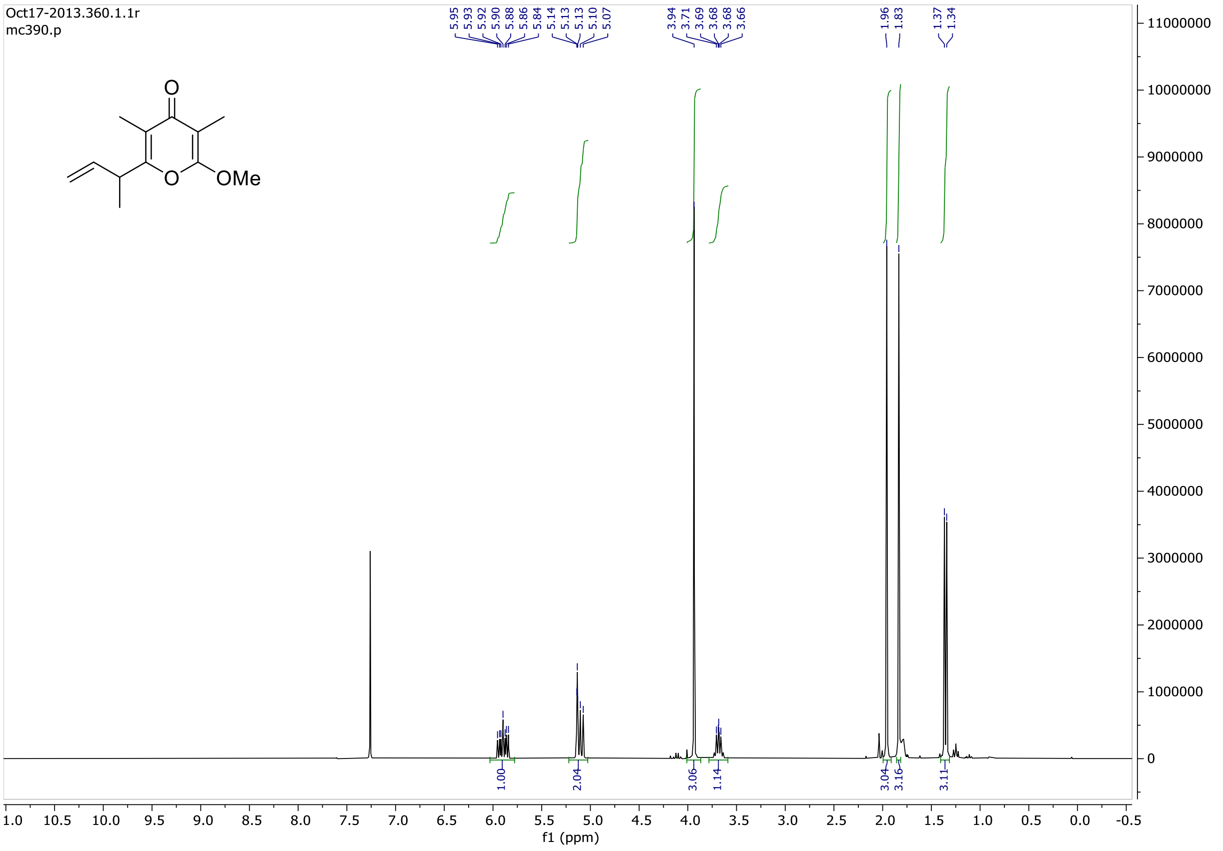

[13]C (75Mz, $CDCl_3$, 293 K) of 8

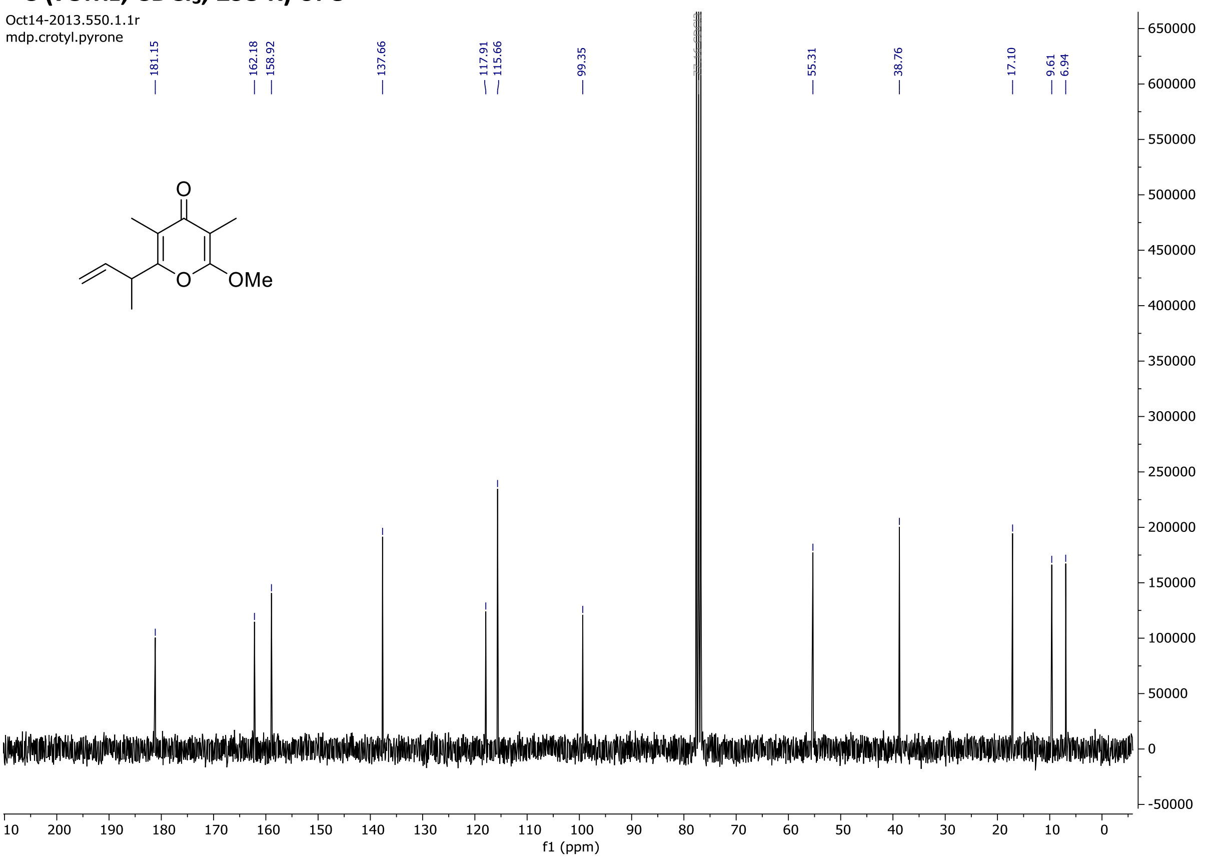

^{1}H (300 MHz, $CDCl_3$, 293 K) of crude 9

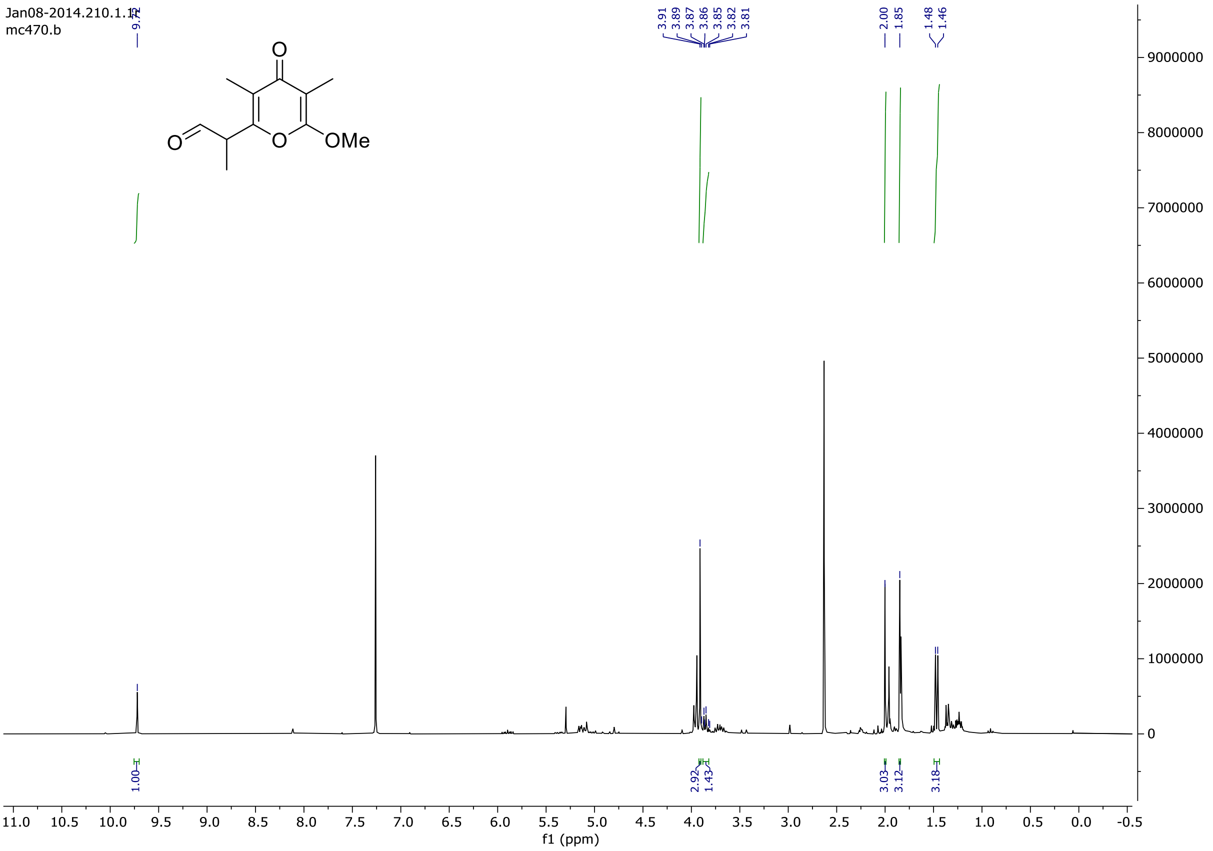

^{1}H (300 MHz, $CDCl_3$, 293 K) of 11

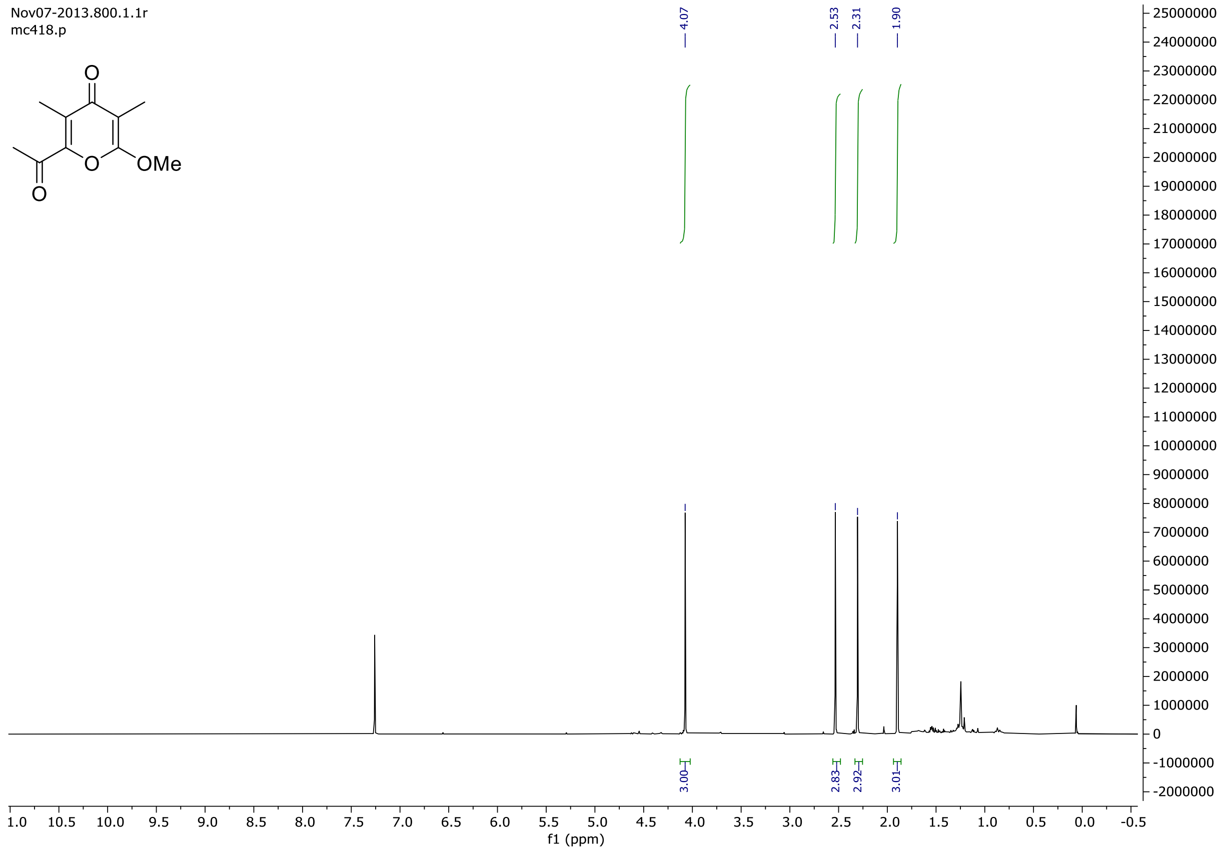

^{13}C (75Mz, $CDCl_3$, 293 K) of 11

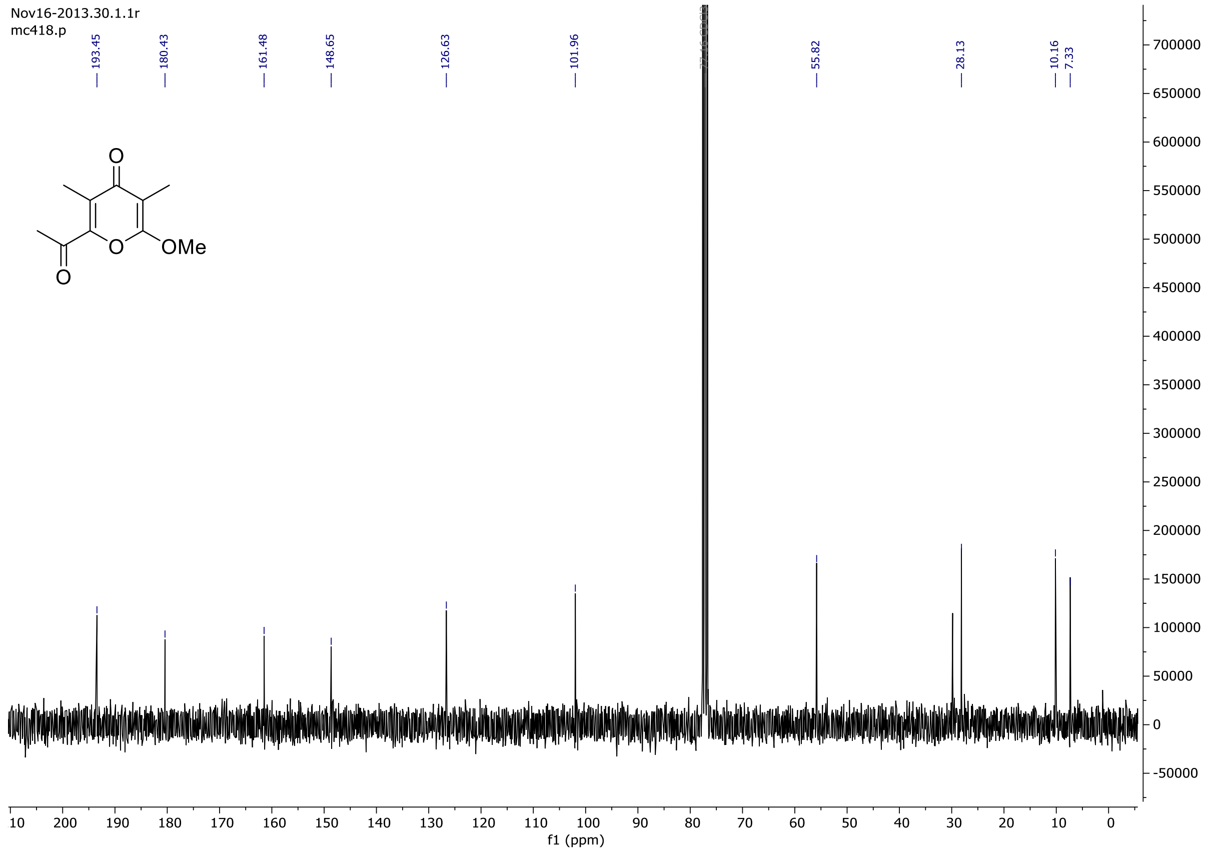

^{1}H (300 MHz, CDCl$_3$, 293 K) of 12

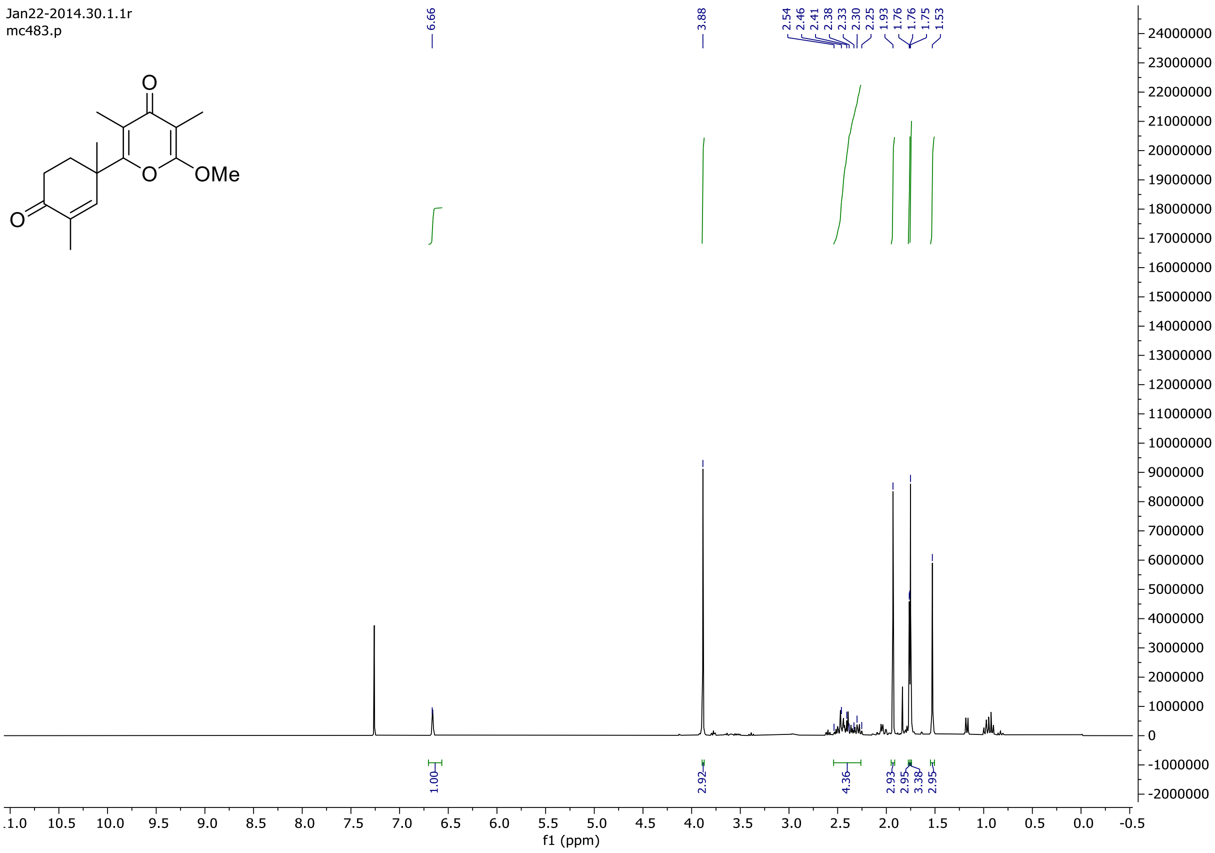

^{13}C (75Mz, CDCl$_3$, 293 K) of 12

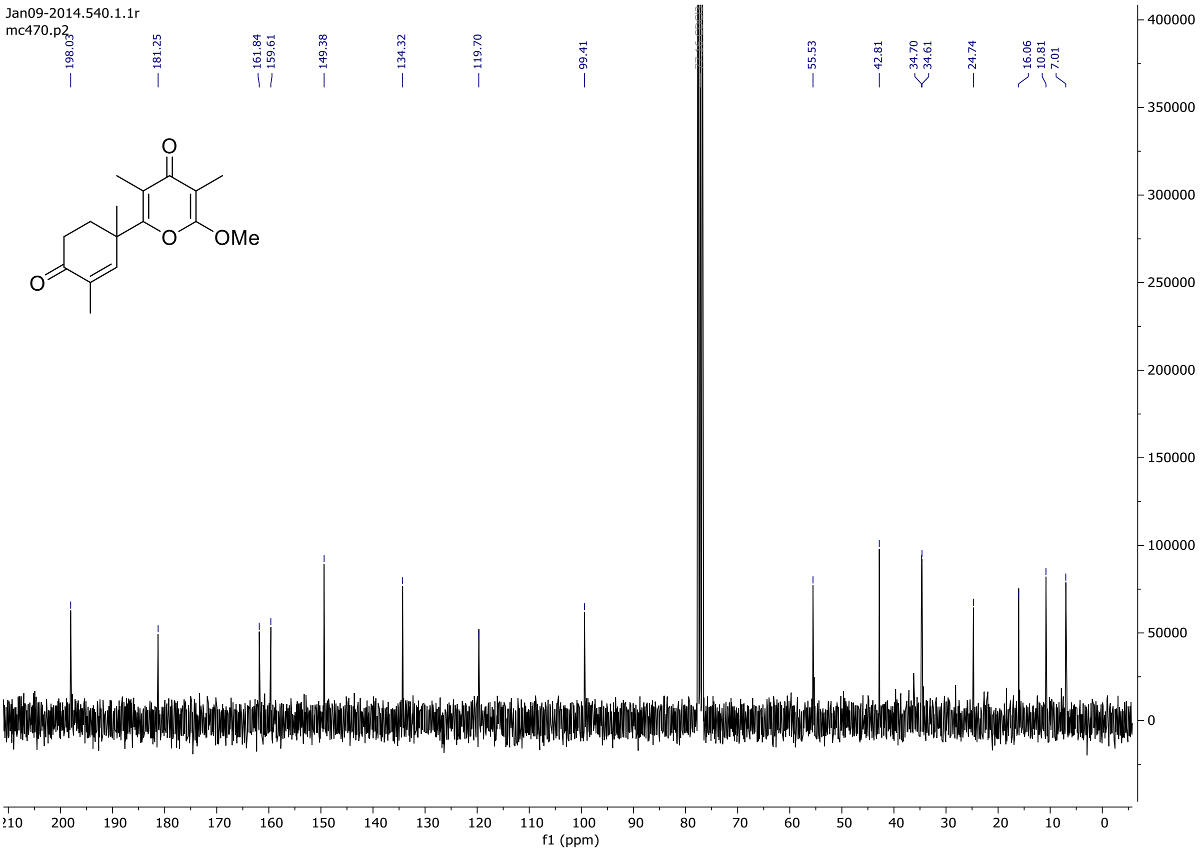

^{1}H (300 MHz, $CDCl_3$, 293 K) of 13 (2 diastereomers)

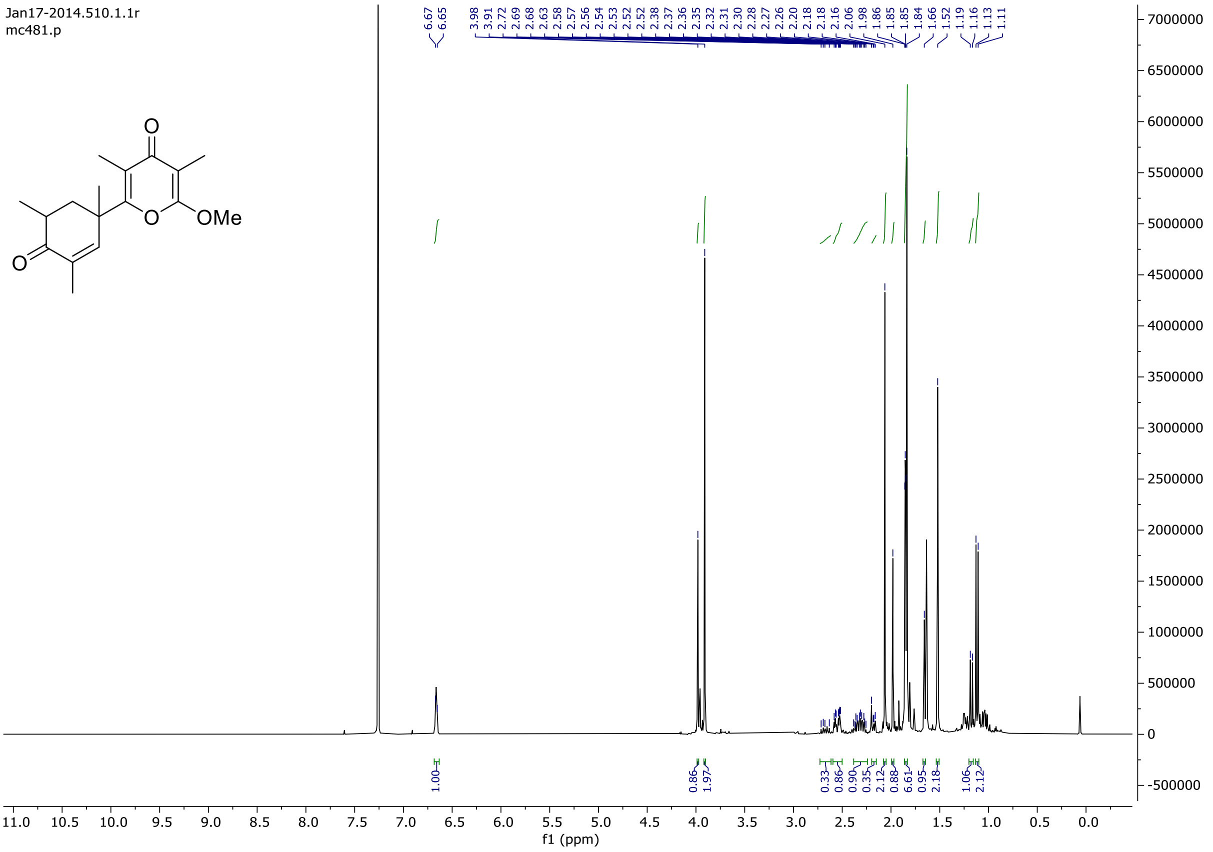

^{13}C (75Mz, $CDCl_3$, 293 K) of 13 (2 diastereomers)

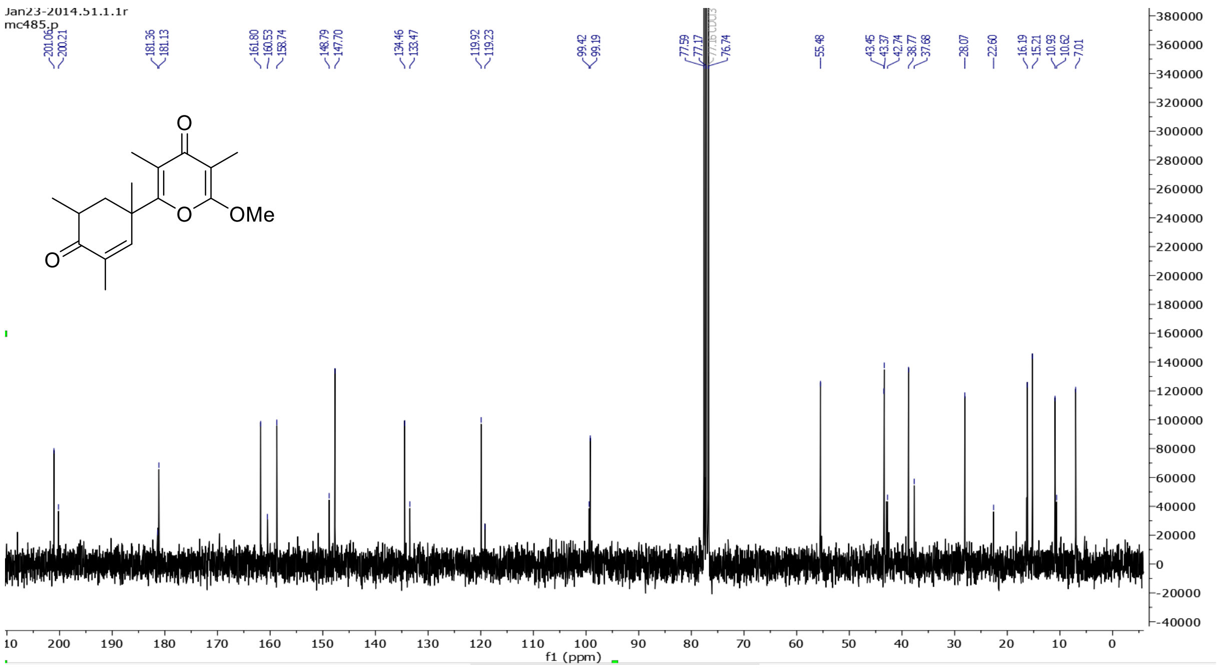

^{1}H (300 MHz, CDCl$_3$, 293 K) of 5

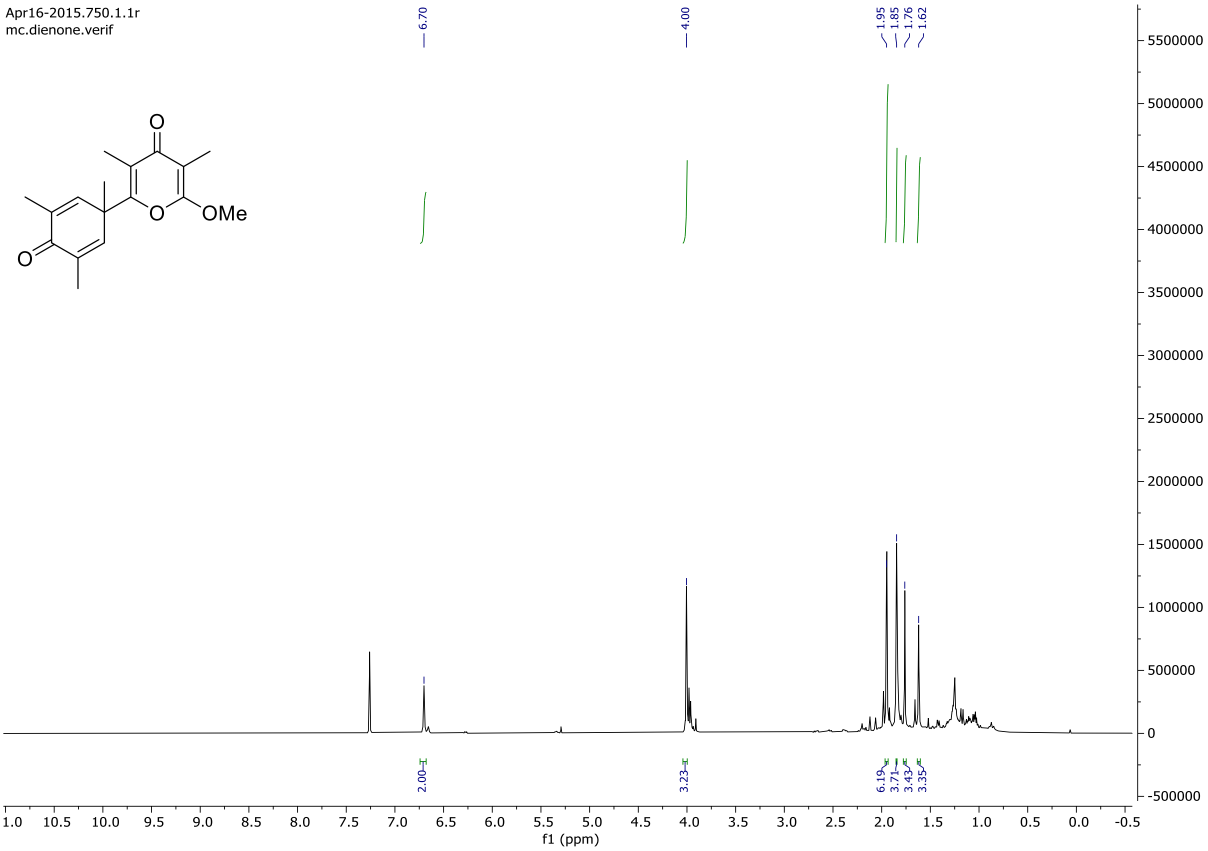

[13]C (75Mz, CDCl$_3$, 293 K) of 5

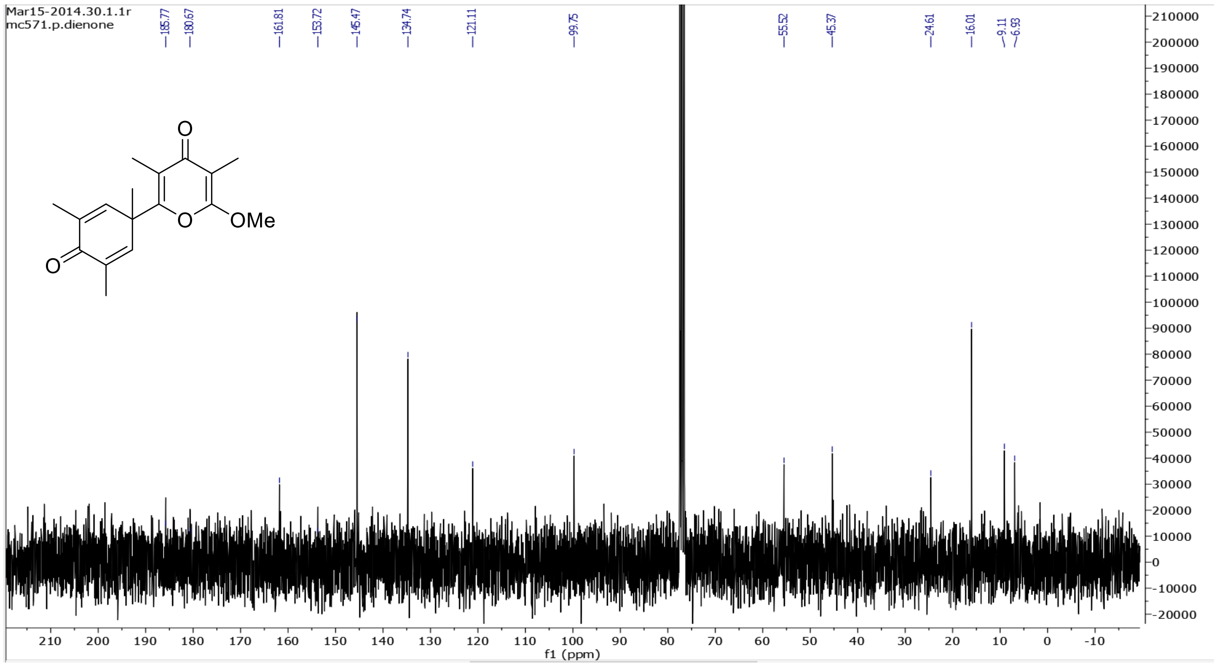

^1H (300 MHz, $CDCl_3$, 293 K) of 14

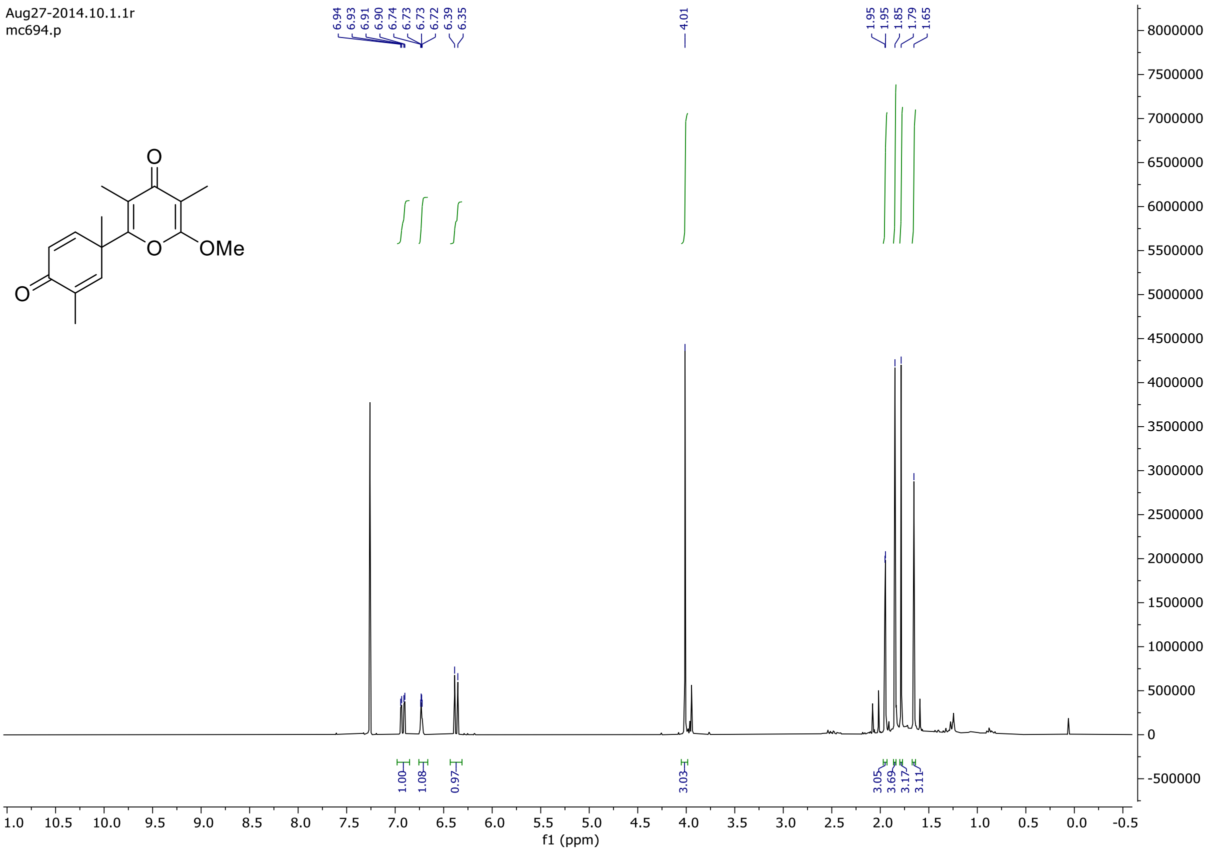

^{13}C (75Mz, $CDCl_3$, 293 K) of 14

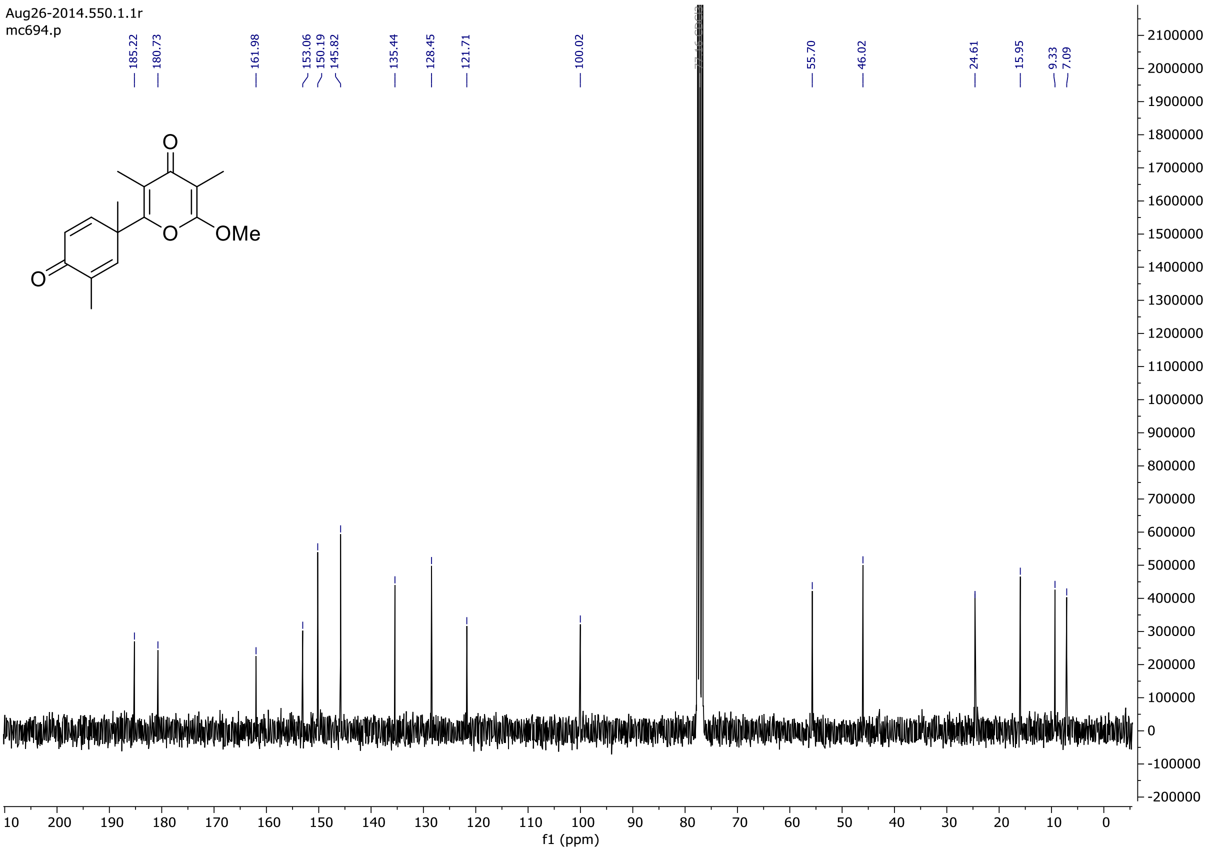

^{1}H (300 MHz, $CDCl_3$, 293 K) of spectra of 17

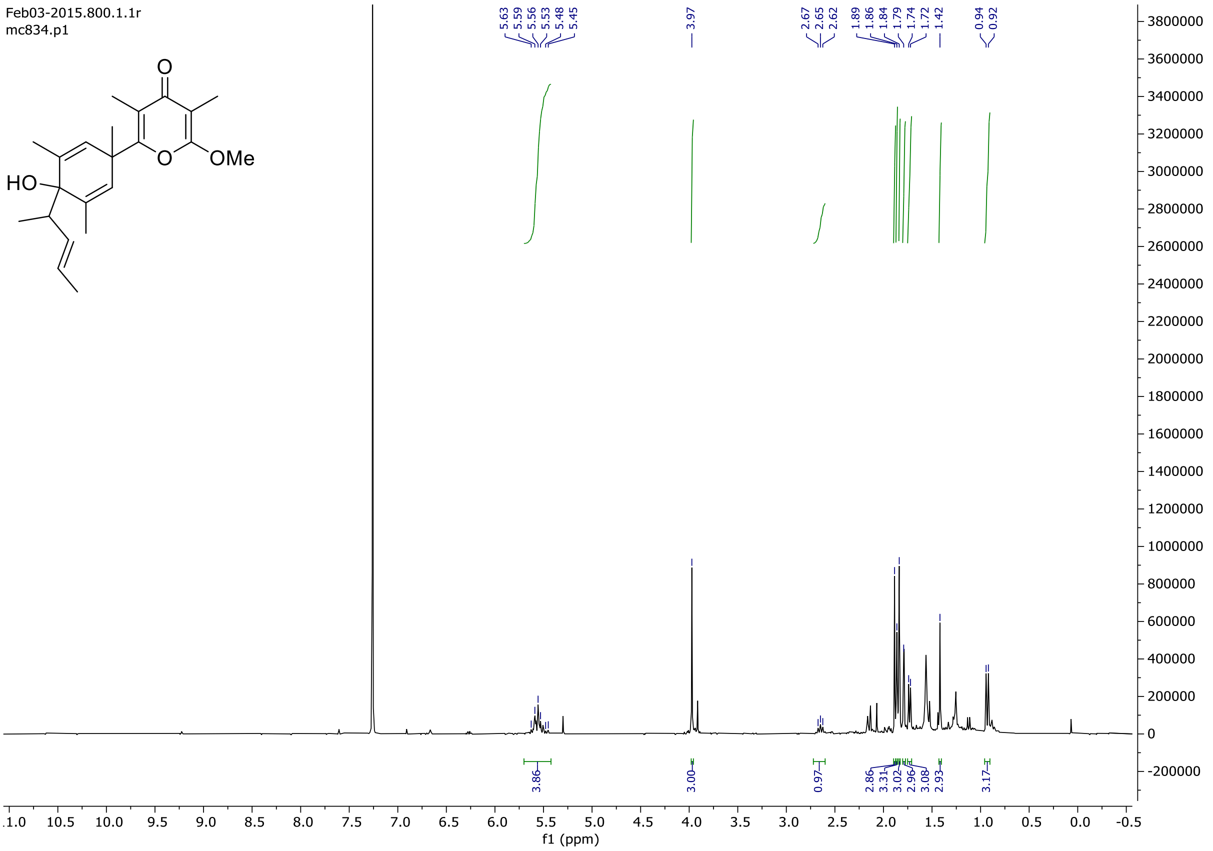

^{13}C (75Mz, $CDCl_3$, 293 K) of 17

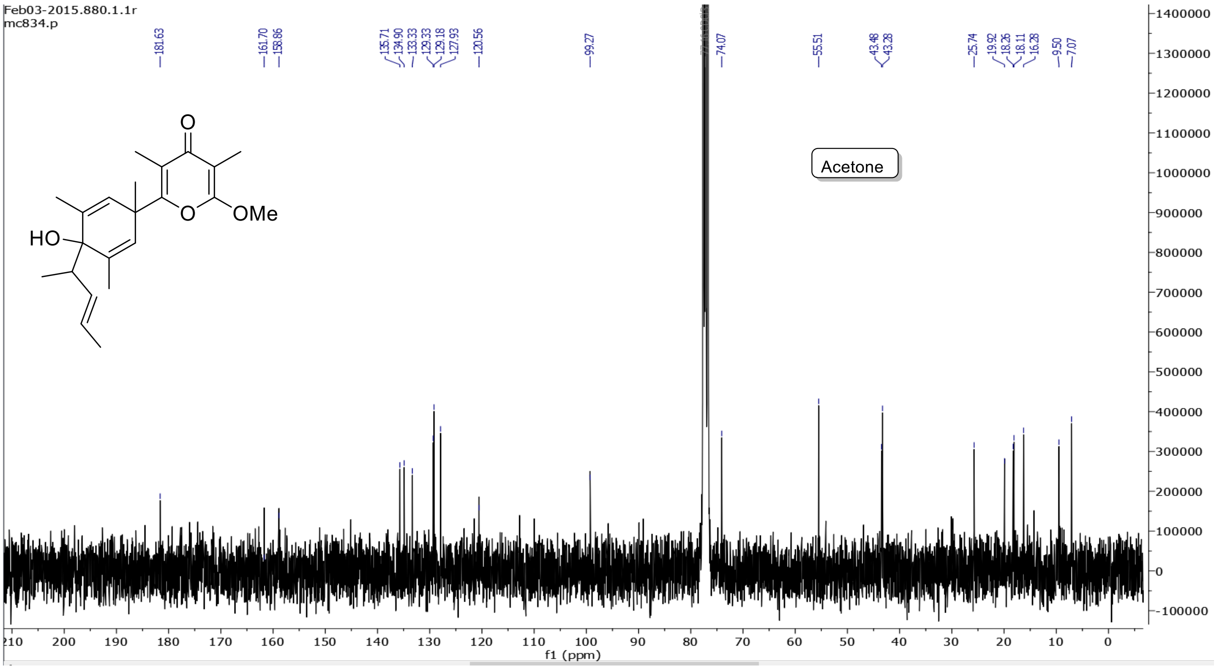

^{1}H (300 MHz, $CDCl_3$, 293 K) of 19

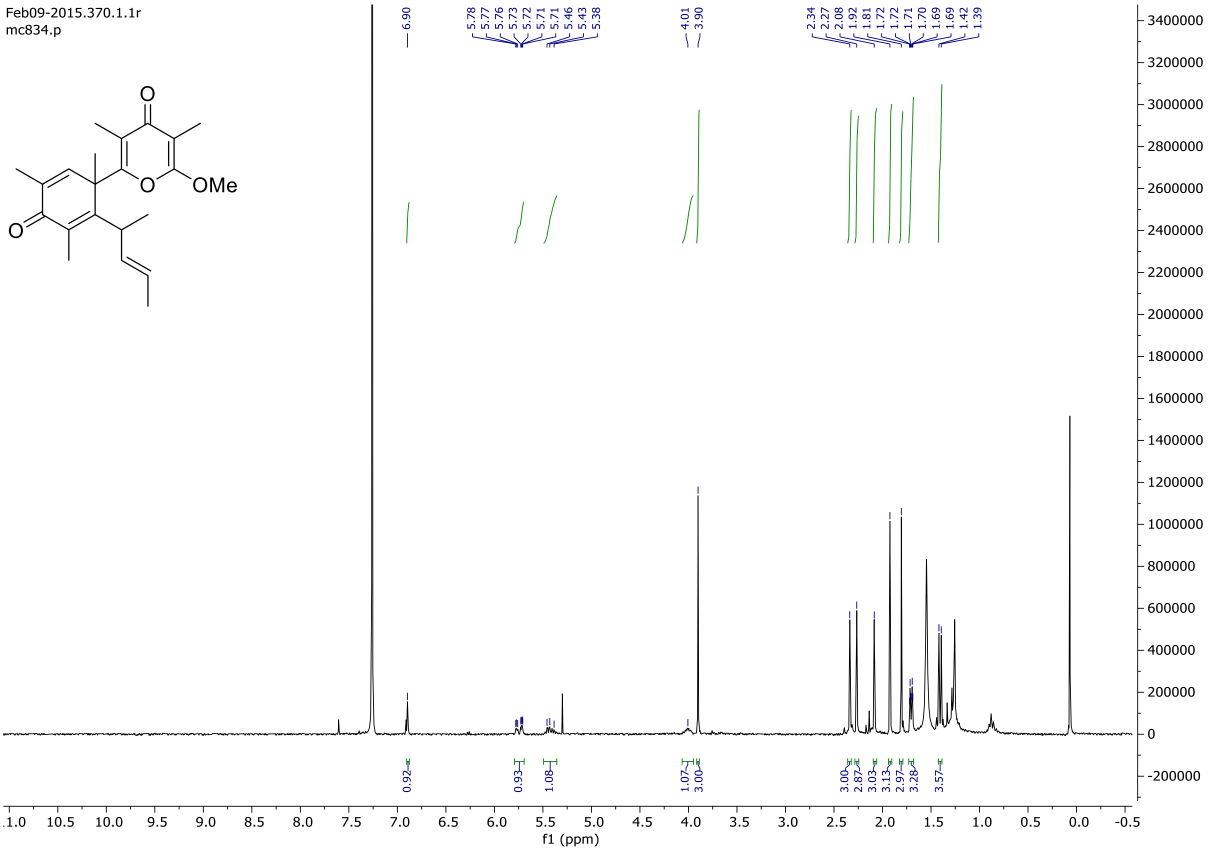

COSY (300 MHz, $CDCl_3$, 293 K) of 19

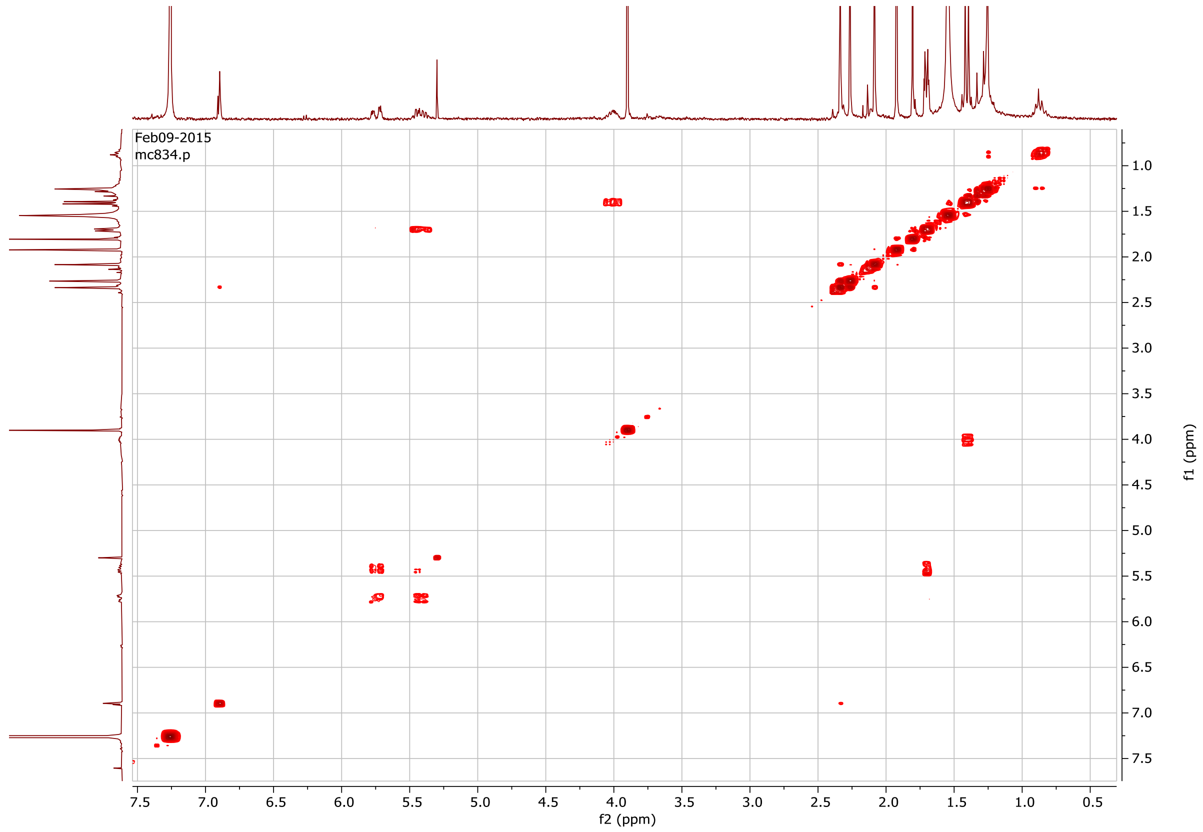

Supplement: File 1 — Experimental details, 1H and 13C spectra of new compounds. [file Beilstein_J_Org_Chem-18-1741-s001.pdf]
